# Structural complexity of the co-chaperone SGTA: a conserved C-terminal region is implicated in dimerization and substrate quality control

**DOI:** 10.1186/s12915-018-0542-3

**Published:** 2018-07-11

**Authors:** Santiago Martínez-Lumbreras, Ewelina M. Krysztofinska, Arjun Thapaliya, Alessandro Spilotros, Dijana Matak-Vinkovic, Enrico Salvadori, Peristera Roboti, Yvonne Nyathi, Janina H. Muench, Maxie M. Roessler, Dmitri I. Svergun, Stephen High, Rivka L. Isaacson

**Affiliations:** 1 Department of Chemistry, King’s College London, Britannia House, Trinity Street, London, SE1 1DB UK; 2 European Molecular Biology Laboratory, Hamburg Outstation, Notkestrasse 85, 22603 Hamburg, Germany; 3 Department of Chemistry, University of Cambridge, Lensfield Road, Cambridge, CB2 1EW UK; 4 School of Biological and Chemical Sciences, Queen Mary University of London, Mile End Road, London, E1 4NS UK; 5 London Centre for Nanotechnology, University College London, 17-19 Gordon Street, London, WC1H 0AH UK; 6 School of Biological Sciences, Faculty of Biology, Medicine and Health, University of Manchester, Manchester Academic Health Science Centre, The Michael Smith Building, Oxford Road, Manchester, M13 9PT UK; 7 Present Address: School of Life Sciences, University of Lincoln, Joseph Banks Laboratories, Green Lane, Lincoln, LN6 7DL UK

## Abstract

**Background:**

Protein quality control mechanisms are essential for cell health and involve delivery of proteins to specific cellular compartments for recycling or degradation. In particular, stray hydrophobic proteins are captured in the aqueous cytosol by a co-chaperone, the small glutamine-rich, tetratricopeptide repeat-containing protein alpha (SGTA), which facilitates the correct targeting of tail-anchored membrane proteins, as well as the sorting of membrane and secretory proteins that mislocalize to the cytosol and endoplasmic reticulum-associated degradation. Full-length SGTA has an unusual elongated dimeric structure that has, until now, evaded detailed structural analysis. The C-terminal region of SGTA plays a key role in binding a broad range of hydrophobic substrates, yet in contrast to the well-characterized N-terminal and TPR domains, there is a lack of structural information on the C-terminal domain. In this study, we present new insights into the conformation and organization of distinct domains of SGTA and show that the C-terminal domain possesses a conserved region essential for substrate processing in vivo.

**Results:**

We show that the C-terminal domain region is characterized by α-helical propensity and an intrinsic ability to dimerize independently of the N-terminal domain. Based on the properties of different regions of SGTA that are revealed using cell biology, NMR, SAXS, Native MS, and EPR, we observe that its C-terminal domain can dimerize in the full-length protein and propose that this reflects a closed conformation of the substrate-binding domain.

**Conclusion:**

Our results provide novel insights into the structural complexity of SGTA and provide a new basis for mechanistic studies of substrate binding and release at the C-terminal region.

**Electronic supplementary material:**

The online version of this article (10.1186/s12915-018-0542-3) contains supplementary material, which is available to authorized users.

## Introduction

**Fig. 1:**
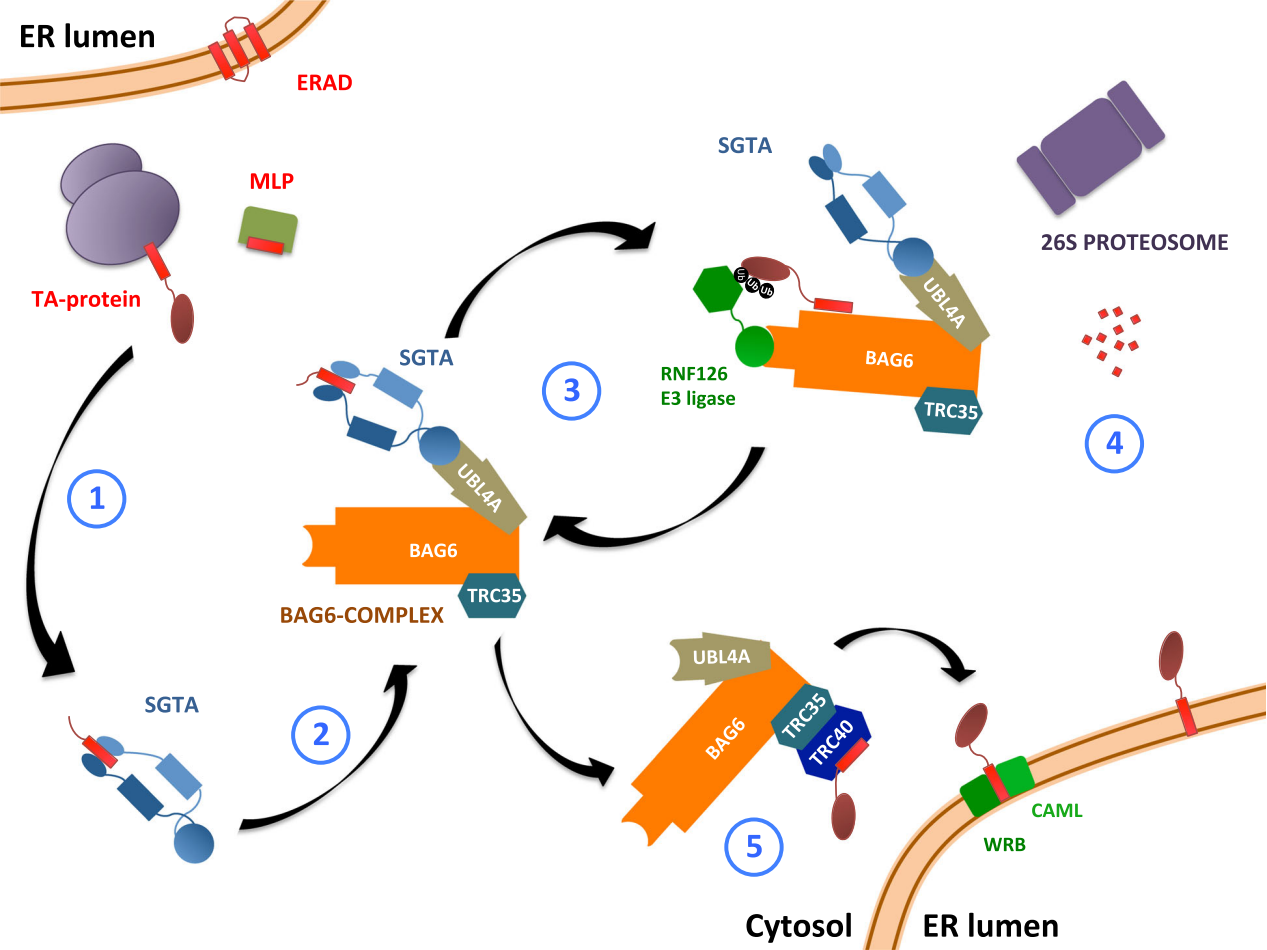
Roles of SGTA in cytoplasmic quality control. SGTA binds to hydrophobic substrates (MLPs or TMDs of TA proteins) via its C-terminal domain (1). It collaborates with the BAG6 complex (composed of BAG6, TRC35, and UBL4A) which interacts with SGTA through its UBL domains (2). Hydrophobic substrates bound to the BAG6 complex can be ubiquitinated by E3 ligase RNF126 (3) and targeted for degradation via the UPS (4). SGTA TPR domain interacts with the proteasomal subunit RPN13. SGTA shields exposed hydrophobic regions on TA proteins en route to ER membrane insertion, handing them on to the downstream TRC40 targeting complex (5)

Homo- and hetero-multimerization of proteins can confer many benefits on a system including orientating different binding partners into suitable proximity, increasing local concentrations of reactants, and capturing substrates with tweezer- or scissor-like actions [1]. Multimerization is particularly useful in mechanisms that rely on cascades of transient, low affinity interactions, such as the collaboration between co-chaperone SGTA (small glutamine-rich, tetratricopeptide repeat-containing protein alpha) and the BAG6 complex [2], which regulates the fate of hydrophobic proteins exposed to the aqueous environment of the cytoplasm in cells (summarized in Fig. 1). These substrates include misfolded or mislocalized membrane and secretory proteins (MLPs) [3] that are ultimately destined for proteasomal degradation and tail-anchored (TA) proteins that are en route for TRC40-mediated targeting to the ER. In the case of TA proteins, the C-terminal location of their ER targeting signal necessitates a post-translational delivery mechanism [4].

The heterotrimeric BAG6 complex is thought to be composed of a dimer or multimer of heterotrimers [5], each comprising one copy of BAG6 (BCL2-associated athanogene 6), TRC35 (transmembrane recognition complex 35), and UBL4A (ubiquitin-like protein 4A). BAG6, for which limited structural information exists, is a 119 kDa protein whose oligomerization is not yet well defined [6]. The non-canonical “BAG domain” region close to the C-terminus of BAG6 is, however, known to bind directly to a C-terminal region of UBL4A with crystal structures of the minimal complex solved [5, 7]. Similarly, a C-terminal stretch of BAG6, adjacent to the “BAG domain” interacts with the C-terminal domain of TRC35 [3].

The ability of SGTA to interact with the BAG6 complex and a common cohort of hydrophobic substrates enables it to transfer selected protein clients onto the BAG6 complex for sorting and triage [8, 9]. Potential outcomes may include refolding to a native conformation, targeting to the correct subcellular destination or selective degradation [10–13]. Hence, hydrophobic substrates, such as MLPs, bound to the BAG6 complex can be ubiquitinated by the E3 ligase, RNF126, and targeted for proteasomal degradation [14, 15]. It is proposed that SGTA can promote substrate deubiquitination, thereby delaying the, normally efficient, degradation of such MLPs and antagonizing the actions of the BAG6 complex [10, 13]. Furthermore, recent studies show that the perturbations of MLP quality control observed upon SGTA overexpression require the binding of its central TPR domain to the intrinsic proteasomal ubiquitin receptor Rpn13 [12, 16].

One well-established role of SGTA is the post-translational targeting of TA proteins to the endoplasmic reticulum (ER) for membrane insertion. Acting as a co-chaperone for the BAG6 complex, SGTA facilitates the transfer of newly synthesized TA proteins to the downstream targeting factor TRC40, also a homodimer but with well-characterized “open” and “closed” states that are regulated via its ATP hydrolysis cycle [17, 18]. TRC40 interacts with C-terminal hydrophobic regions of TA proteins [7, 19] and promotes their delivery to and insertion into the ER membrane via a heterodimeric membrane protein receptor composed of the WRB and CAML proteins [20–22].

SGTA is a structurally unusual homodimer [23] with each 34 kDa monomer consisting of three structurally distinct domains that have interconnected functions. The structure of the N-terminal dimerization domain has been extensively characterized [8, 9, 24] providing insights into its role in TA membrane protein insertion and its interaction with the BAG6 complex in MLP quality control pathways. This region can bind to two different ubiquitin-like domains (UBLs) that are displayed by the BAG6 complex, which constitute the N-terminal domains of both the UBL4A and BAG6 subunits. The central region of SGTA is the most conserved domain, consisting of three, almost identical, tetratricopeptide repeats (TPRs) arranged in tandem, each formed by a pair of α-helices folded in an antiparallel fashion [25]. The structure of the SGTA TPR domain was determined previously by X-ray crystallography, and its three TPRs are followed by a C-terminal capping helix [25]. This domain has also been reported to interact directly with Hsp70 and Hsp90 chaperones [2, 26] and the proteasomal subunit Rpn13 [12, 27]. The TPR domain has also been linked to viral replication and hormone receptor signaling, with implications for both health and disease [28, 29]. The C-terminal substrate-binding domain of SGTA contains a glutamine-rich stretch [30] and is known to bind hydrophobic substrates [31] but its structure is, as yet, completely undetermined.

Studies of the C-terminal domain of SGTA have thus far focused on the glutamine-rich region, and the role of the well-conserved remainder has not yet been explored [30]. In this work, we identify a region of the SGTA C-terminal domain that alters its impact on MLP quality control. Moreover, via an integrated biophysical analysis, we explore the structural and dynamic properties of full-length SGTA in vitro. We demonstrate that the C-terminal domain can exist in solution as a dimer, and provide the first evidence that SGTA exhibits a C-terminally “closed” conformation stabilized by an NNP region that is important for the efficient processing of mislocalized proteins.

## Results

### The NNP region in the C-terminal domain contributes to SGTA function

**Fig. 2:**
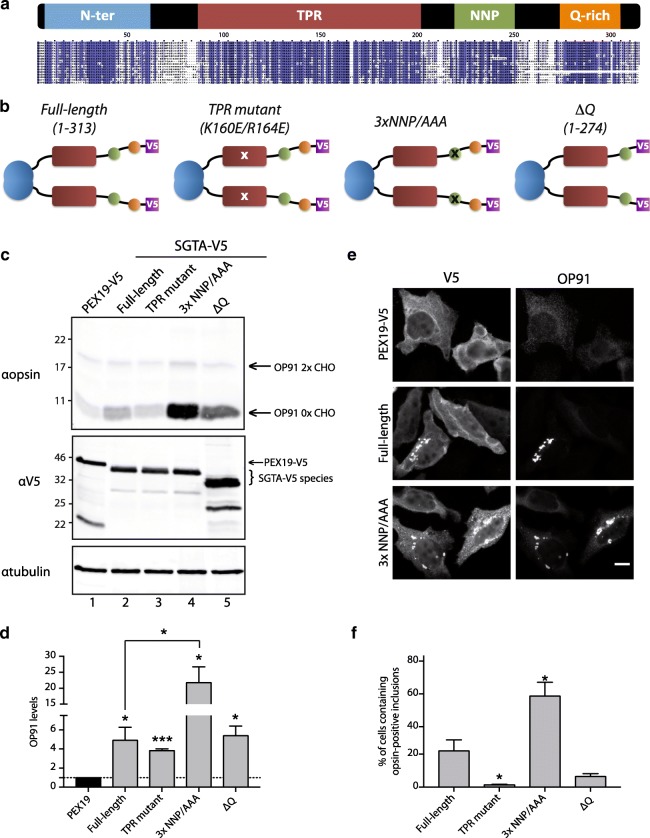
Mutations in SGTA NNP region enhance the steady state of a model MLP and promote its accumulation in cytoplasmic inclusions. **a** Schematic representation of SGTA domain organization alongside a sequence alignment (purple indicates high conservation; expanded version of the alignment is shown in Additional file 1: Figure S1). **b** Summary of all SGTA-V5 variants designed for this part of the work. **c** HeLa cells stably expressing OP91 under an inducible promoter were transiently transfected with plasmids encoding either a control (PEX19-V5) or V5-tagged SGTA variants as indicated, and OP91 expression induced. The resulting levels of OP91 and exogenous PEX19/SGTA variants were visualized via Western blotting and fluorescence based detection (LI-COR). **d** Quantified signals for PEX19-V5 or SGTA-V5 species were normalized to endogenous tubulin, which acted as a loading control and the OP91 levels then expressed relative to the respective normalized V5 signal, with the ratio for OP91/PEX19-V5 set as one. Values show means ± s.e.m. from three independent technical repeats; *P* was determined by a Student’s *t* test. **e** HeLa cells stably expressing OP91 were co-transfected as in (**c**), the cells fixed, and then labeled for immunofluorescence microscopy using antibodies that recognize either OP91 or the V5 tag on PEX19 and the SGTA variants. Scale bar is 10 μm (see also Additional file 2: Figure S2). **f** The percentage of cells displaying opsin-positive inclusions were counted from the experiments described in (**e**) (*n* ≥ 100 cells/condition from total of three experiments). Error bars show s.e.m., and *P* is determined by a Student’s *t* test

Based on sequence homology (Fig. 2a and Additional file 1: Figure S1), we propose that SGTA contains four well-defined regions of conservation: the first two regions match the widely studied N-terminal and TPR domains, respectively, while we have sub-divided the remaining C-terminal section in two: a region characterized by three repetitions of a NNP motif (that we call the NNP region), followed by a separate glutamine-rich stretch (Q-rich region) (Fig. 2a). While the Q-rich region has previously been implicated in binding hydrophobic substrates [30], the potential role of the NNP motifs has not been addressed. We therefore prepared a mutant version of SGTA-V5 [13] in which all three NNP motifs were altered to triple alanines (SGTA-3xNNP/AAA-V5), together with a truncated form of SGTA that retained the NNP motifs but lacked the Q-rich region (SGTA-ΔQ-V5; see Fig. 2b).

SGTA participates in the selective quality control of mislocalized membrane proteins (MLPs) that have failed to reach the endoplasmic reticulum (ER) and defaulted to the cytosol [12, 13, 32]. The overexpression of exogenous SGTA can lead to an increase in the steady-state level of such MLPs, most likely by perturbing their otherwise efficient proteasomal degradation [13, 33]. We therefore asked how the mutation of the NNP motifs and loss of the Q-rich region affected the steady-state level of OP91, an N-terminal fragment of the polytopic integral membrane protein opsin, which acts as a model MLP in cultured mammalian cells [13, 27]. When SGTA-3xNNP/AAA-V5 was overexpressed in HeLa cells induced to produce OP91, a clear increase in the amount of cytosolic, non-glycosylated, OP91 was seen as compared to the expression of a PEX19 control (Fig. 2c). This > 20 fold increase was well above the ~ 5 fold increase seen with full-length SGTA-V5 [12] (Fig. 2c, d). In contrast, the normalized effect of SGTA-ΔQ-V5 co-expression on OP91 levels was comparable to that observed using SGTA-V5 (Fig. 2d) indicating that deleting just the Q-rich region had no measurable effect.

In addition to enhancing the steady-state level of model MLPs including OP91, the co-expression of exogenous SGTA-V5 results in the appearance of discrete cytosolic inclusions that contain both SGTA and model MLP substrates [13]. We therefore transiently expressed SGTA variants, or the PEX19 control, in HeLa cells induced to make OP91 and analyzed them by immunofluorescence microscopy. In agreement with the increase in steady-state OP91 levels (Fig. 2c, d), the number of intracellular inclusions that contain OP91 was greater in the presence of the SGTA-3xNNP/AAA-V5 mutant as compared to wild-type SGTA-V5 (Fig. 2e, f and Additional file 2: Figure S2). In contrast, the previously described SGTA TPR mutant [12], which is unable to interact with chaperones or the proteasome, showed a clear reduction in the number of intracellular OP91 containing inclusions (Fig. 2f and Additional file 2: Figure S2), while the effect of the SGTA-ΔQ-V5 mutant was not statistically significant (Fig. 2f and Additional file 2: Figure S2). Quantification revealed that the number of cells containing large perinuclear inclusions was threefold greater in the presence of SGTA-3xNNP/AAA-V5 than SGTA-V5 (Fig. 2f). Interestingly, although exogenous SGTA-V5 only enters cytosolic inclusions in the presence of an MLP substrate (Additional file 3: Figure S3), as previously reported [13], the SGTA-3xNNP/AAA-V5 variant can form these structures in the absence of OP91 induction (Additional file 3: Figure S3). On the basis of these findings, we concluded that the NNP motifs in the C-terminal region of SGTA make a distinct contribution to its cellular function and set out to determine their structural significance.

### The C-terminal domain of SGTA is a partially folded region with the ability to dimerize

**Fig. 3:**
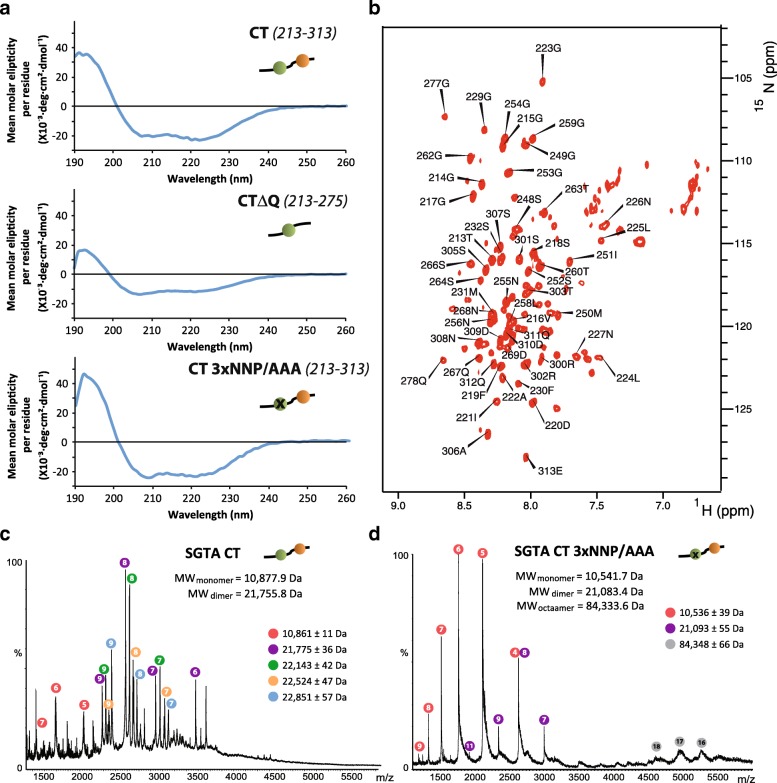
Biophysical characterization of SGTA C-terminal domain. **a** Far UV CD spectra of the C-terminal domain of SGTA (top), its version lacking the glutamine-rich region (middle) and the C-terminal mutated in the NNP region (bottom). **b** ^1^H-^15^N TROSY HSQC of the deuterated version of SGTA C-terminal domain with partial assignment. **c**, **d** Native mass spectrometry spectra of the C-terminal domain of SGTA (**c**) and of the SGTA 3xNNP/AAA C-terminal mutant (**d**). Peaks corresponding to the same species are marked with colored circles with the charge number written inside. The theoretical molecular weight appears under the title and the obtained molecular weights for each species are shown on the right in the same color scheme

Having demonstrated that the conserved NNP region at the C-terminus of SGTA contributes to its cellular function(s), we performed structural and biophysical studies to better define this domain. In the first instance, we produced both the complete C-terminal domain (CT; residues 213–313) and a version lacking the Q-rich region (CTΔQ; residues 213–274). Circular dichroism (CD) analysis of both fragments in the far UV region of the spectra showed characteristic α-helical minima of elipticity at 208 and 222 nm and a maximum around 190 nm, features that were also retained in the CT construct in which the three NNP motifs were mutated to AAA (Fig. 3a). A deconvolution analysis of the spectra showed around 46 and 36% of α-helical secondary structure for the CT and the CTΔQ proteins, respectively.

The NMR ^1^H-^15^N HSQC spectrum of the C-terminal domain showed poor peak dispersion which is characteristic of unfolded protein (Fig. 3b). Despite the extensive optimization of buffer, temperature and pH conditions (see the “Methods” section and Additional file 4: Figure S4), we were unable to complete the backbone assignment (BMRB accession number: 27272) due to a severe line broadening effect present in two regions of the sequence representing ~ 20 residues in the NNP area and ~ 30 residues in the Q-rich region. The CTΔQ version presented identical problems for the same NNP residues (Additional file 5: Figure S5). Chemical shift index (CSI) analysis (Cα, Cβ, and CO) of the assignable regions predominantly indicated random coil except for those residues immediately surrounding the missing regions, which presented α-helical propensity (Additional file 6: Figure S6) suggesting that the unassigned regions account for the helical content observed by CD.

The C-terminal domain of SGTA showed a tendency to aggregate, eluting far earlier than expected in size-exclusion chromatography and displaying aggregation propensity in dynamic light scattering (DLS) experiments (Additional file 7: Figure S7). In addition, we have observed that protein samples at high concentration undergo a phase transition from solution to hydrogel.

In order to further investigate its oligomerisation state, we performed native mass spectrometry experiments on the SGTA CT protein. Surprisingly we found that the major species present in solution is a dimer (Fig. 3c). When these data were analyzed in more detail, we detected different forms of the C-terminal dimer that vary in molecular weight by multiples of 359 ± 23 Da. We have discounted covalent modifications of the protein by performing mass spectrometry on the same protein in denaturing conditions, which no longer exhibits this additional mass (Additional file 8: Figure S8). We conclude that these larger species most likely correspond to a non-covalently bound molecule present at a stoichiometry of between one and four times that of the SGTA C-terminal region. Because the C-terminal region of SGTA is proposed to recognize hydrophobic substrates [30], we speculate that this molecule might be a bacterial lipid or hydrophobic tripeptide derived from the recombinant expression in *Escherichia coli*. However, all our attempts to identify this molecule have thus far proved inconclusive.

To mimic our biological experiments, we performed equivalent MS on the C-terminal 3xNNP/AAA mutant. In this case, the major species was the monomer with a minor representation of aggregated states and no additional mass was present (Fig. 3d). The propensity of this mutant to aggregate was also observed in analytic SEC (Additional file 7: Figure S7C). We conclude that mutating the NNP region in this way strongly destabilizes the C-terminal dimerization.

### SGTA domains behave as structurally independent units

**Fig. 4:**
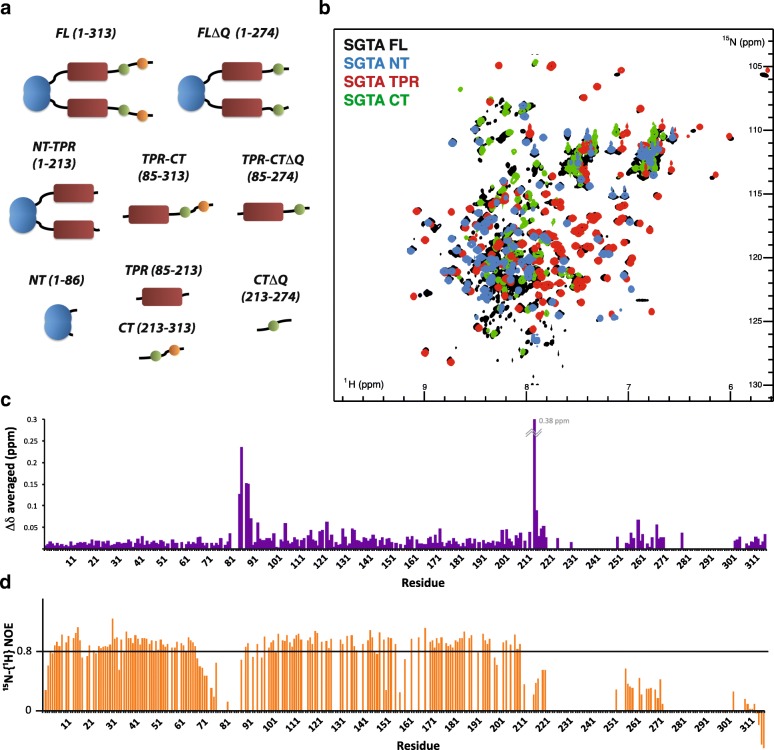
NMR analysis of SGTA constructs. **a** Cartoon representation of the different SGTA variants used for NMR comparison. **b** Overlaid ^1^H-^15^N HSQC spectra of FL SGTA (black), NT (blue), TPR (red), and CT (green) constructs. **c** Chemical shift difference analysis of amide signals from N-terminal, TPR, and C-terminal region in the single domain context versus FL SGTA. **d** ^15^N - {^1^H} heteronuclear NOE measurements for FL SGTA

Having established that the C-terminal region of SGTA is able to dimerize when expressed as an excised polypeptide, we wondered how such an interaction would affect the overall domain arrangement of SGTA. We acquired a set of ^1^H-^15^N HSQC spectra for a series of SGTA-derived polypeptides (Fig. 4a, b) representing a wide range of domain combinations, under identical conditions. For the proteins containing the Q-rich region of SGTA (FL and CT; Fig. 4a), deuteration was required because of a major peak broadening effect probably due to the C-terminal aggregation tendency. All construct spectra were assigned using some previously obtained data (BMRB accession numbers: 19779 and 5709) and additional 3D backbone experiments (see the “Methods” section). We achieved 84% total backbone assignments for FL SGTA with only the aforementioned missing NNP and Q-rich regions left unassigned (CT, TPR-CTΔQ, and NT assignments were deposited as BMRB accession numbers: 27272, 27275 and 27276, respectively).

The HSQC spectral comparison shows that the fold of each domain is maintained across all constructs analyzed (Fig. 4b and Additional file 5: Figure S5); hence, almost no change is observed upon chemical shift value analysis for each amino acid, except in the boundary regions between the domains (Fig. 4c and Additional file 9: Figure S9). These results suggest that each domain is structurally independent of the presence of the rest of the protein and that there are minimal inter-domain contacts. They also indicate that the C-terminal dimerization, if it occurs in the longer constructs, is not perturbing the chemical shift values of the rest of the protein. The chemical shift differences between the CT and CTΔQ constructs (Additional file 8: Figure S8) are more significant than in all the other comparisons and extend beyond the residues close to the truncation point. This points to stabilizing interactions between the Q-rich region and the rest of the C-terminal domain.

Finally, we used ^15^N - {^1^H} heteronuclear NOE measurements of the different SGTA derivatives to address the motion of NH vectors for each amino acid (Fig. 4d and Additional file 10: Figure S10). The results confirmed the previously defined folded domains and unstructured linkers of the N-terminal dimerization and TPR domains. Beyond the TPR domain, the majority of heteronuclear NOE values are typical of flexible polypeptide, but those obtained for residues surrounding the unassigned NNP and Q-rich regions are somewhat higher, suggesting that the missing parts tend towards a more ordered arrangement, consistent with our idea that they form α-helices (Additional file 5: Figure S5).

### A closed conformation for the full-length SGTA dimer

Having identified dimerization of the SGTA C-terminal region, and knowing that all domains are structurally independent entities, we employed four complementary experiments to explore the possibility that this may reflect a “closed conformation” in dimeric, full-length SGTA.

**Fig. 5:**
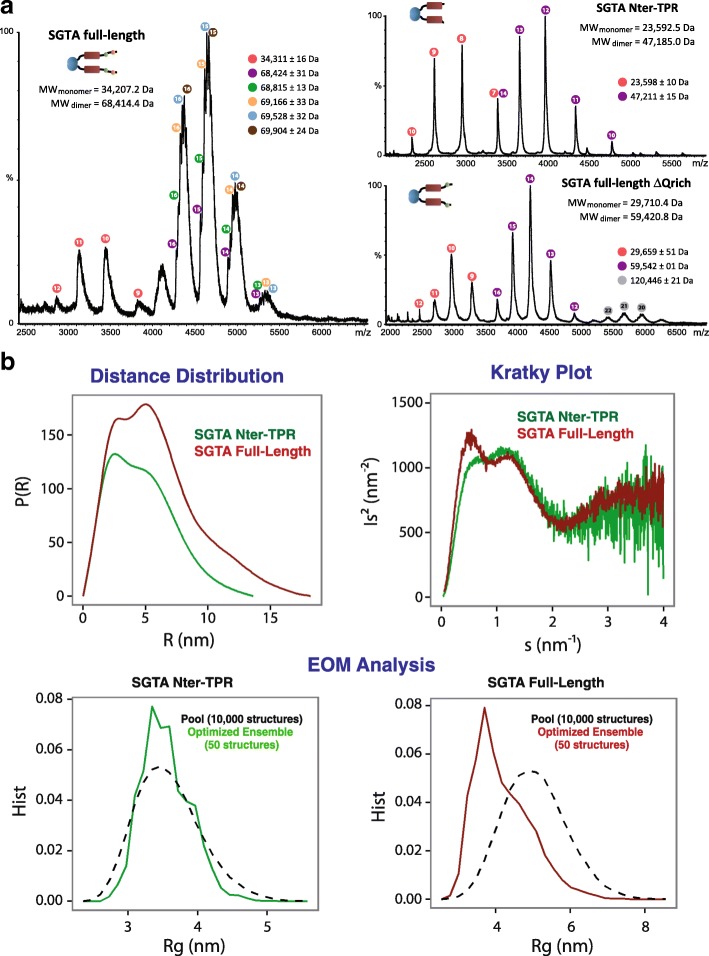
SGTA C-terminal domain is able to dimerise in the full-length context I. **a** Native mass spectrometry chromatograms of the SGTA FL protein (left) and the NT-TPR (upright) and FLΔQ (downright) constructs. Peaks corresponding to the same species are marked with colored circles with the charge number written inside. The theoretical molecular weight appears under the title and the obtained molecular weights for each species are shown on the right following the corresponding color. **b** SAXS analysis of SGTA FL (red) and NT-TPR (green) proteins: distance distribution plot (top left), Kratky plot (top right) and ensemble optimization method analysis data (bottom)

First, using native mass spectrometry, we confirmed that the longer constructs of SGTA containing the N-terminal dimerisation domain are indeed dimers. The results for the NT-TPR, FLΔQ, and FL versions of SGTA clearly showed the dimer as the major species present in solution (Fig. 5). A detailed analysis of the spectra obtained from FL SGTA, again revealed the presence of different variants of the dimer with one to four copies of a similar small molecule bound to it (370 ± 15 Da). This behavior imitates that of the excised C-terminal dimer and was our first indication that the properties we observe with this truncated fragment of SGTA also apply to the full-length protein. Surprisingly, in the construct lacking the Q-rich region, no additional mass was observed suggesting that this region is necessary for binding the presumptive small molecules.

Next, we performed SAXS on full-length SGTA (FL; SASBDB accession number: SASDDB6) and the version lacking the C-terminal domain (NT-TPR variant; SASBDB accession number: SASDDC6) to test whether a possible C-terminal dimerization might constrain the central TPR domain within full-length SGTA. Parameters including the values for radius of gyration (*R*_*g*_), maximum linear dimension (*D*_*max*_), hydrated particle volume (*V*_P_), and the molecular weight (*M*_W_) of the individual constructs are summarized in Table 1. The estimated molecular mass and hydrated particle volume for both proteins indicate that, as expected, they are in a dimeric state (Table 1 and Additional file 11: Table S1), and the peaks in the distance distribution function (Fig. 5b) confirm the presence of multiple domains. The first peak, at about 3 nm, corresponds to distances inside the individual domains, while the second peak, at about 5 nm, corresponds to the inter-domain distance. The fact that this second peak is maintained and becomes even more accentuated with FL SGTA as compared to NT-TPR indicates that the full-length protein adopts a more compact overall conformation in solution.

**Table 1:** Overall parameters estimated from SAXS data: the molecular weight (MW) for each SGTA construct is compared with the expected value for the corresponding dimeric assembly. We report the values of radius of gyration (*R*_g_), the maximum linear dimension (*D*_max_), and the Porod volume (*V*_P_) calculated by the program GNOM

Sample	MW (kDa)	expt. MW (kDa)	*R*_g_ (nm)	*D*_max_ (nm)	*V*_P_ (nm^3^)
NT-TPR	41 ± 4	47	3.6 ± 0.1	14 ± 1	66 ± 1
FL	65 ± 5	68	4.2 ± 0.2	18 ± 1	168 ± 2

The Kratky plots generated from both SGTA constructs show a composite peak structure in the region 0.07–2 nm^−1^, as expected from multi-domain proteins. For *s* > 2 nm^−1^ the Kratky plot tends to a plateau, giving a qualitative indication of moderate flexibility for all the constructs measured. This result is compatible with the presence of flexible portions in both the NT-TPR region and the C-terminal domain (Fig. 5b).

We have also applied an ensemble optimization method (EOM), in order to fit the data in a way that allows for ensembles of possible conformations that may co-exist in solution. In this method, an ensemble of 50 structures is selected to fit the scattering data from a large pool of 10,000 randomized structures. The pool was generated by using the high-resolution structures of N-terminal and TPR domain (4CPG [9] and 2VYI [25] PDB entries, respectively), which are treated as rigid bodies, and modeling the missing portions as dummy residues in random conformations. The results of this EOM approach (Fig. 5b) show that for full-length SGTA, the selected ensemble presents a much lower radius of gyration (*R*_g_) than the average of the structures in the pool, while for the SGTA NT-TPR construct, the selected ensemble has a similar averaged *R*_g_ to that of the pool. This suggests that, in comparison to the NT-TPR region, flexibility is reduced in full-length SGTA, where only a subset of compact conformations fits the data, a situation compatible with a C-terminally closed conformation for SGTA.

**Fig. 6:**
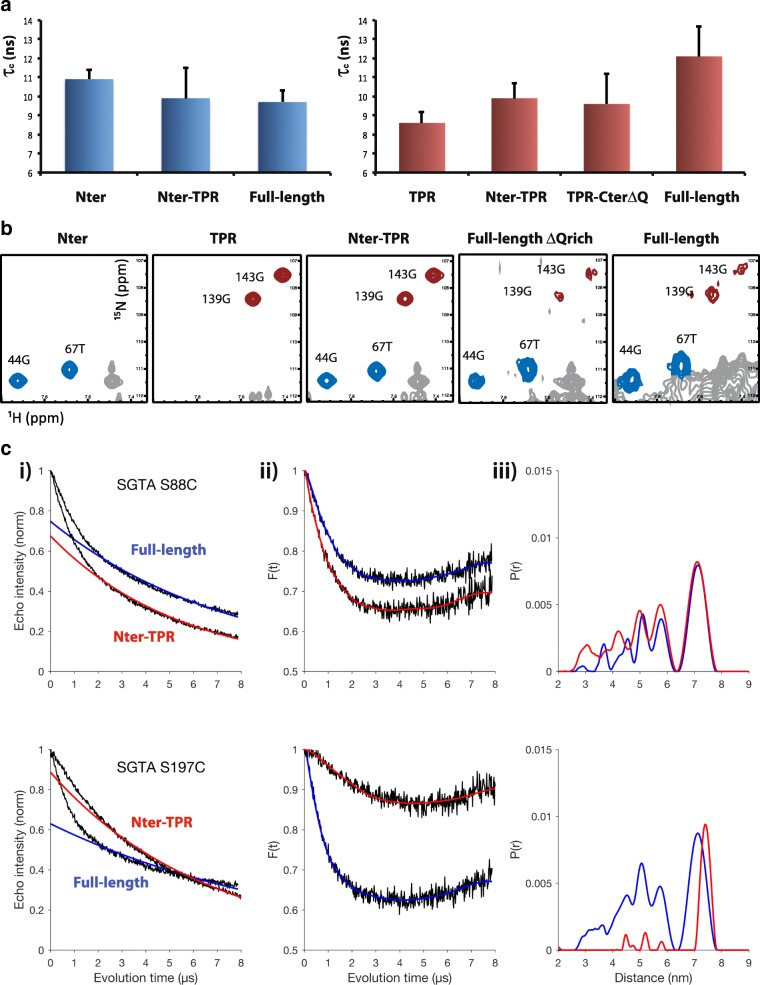
SGTA C-terminal domain is able to dimerise in the full-length context II. **a** Calculated correlations times (τ_c_) for N-terminal (blue) and TPR (maroon) domains in the context of different SGTA constructs (n values and further data in Additional file 12: Figure S11). **b** Detail of ^1^H-^15^N HSQC spectra for different SGTA constructs showing two amide signals from the N-terminal domain (blue) and two signals from the TPR domain (maroon). Note severe broadening effect of TPR signals in FLΔQ and FL constructs. **c** DEER measurements and distances determined for FL and NT-TPR SGTA constructs spin labeled in S88C and S197C mutants. (i) Primary frequency-domain DEER data. (ii) Background-corrected dipolar evolution data (black lines) and corresponding fits obtained through DeerAnalysis2016 [49] by Tikhonov regularization. (iii) Distance distributions obtained by Tikhonov regularization. Blue fits: FL SGTA; red fits: NT-TPR construct

To further corroborate the presence of C-terminal dimerization in the full-length version of SGTA, we studied the NMR relaxation parameters of N-terminal and TPR domains in the context of the different SGTA constructs. We measured the *T*_1_ and *T*_2_ relaxation values and the correlation time (*τ*_c_) for each domain in NT, TPR, NT-TPR, TPR-CTΔQ, and FL SGTA (Additional file 12: Figure S11). Data show that the N-terminal dimerization domain has similar correlation time values (10–11 ns) in all contexts. However, while the correlation time for the TPR domain is similar in the TPR, TPR-CTΔQ and NT-TPR contexts (9–10 ns), the value rises to 12 ns when it is situated within full-length SGTA (Fig. 6a). It should be noted that our correlation time calculations were performed using an isotropic model (due to the impossibility of providing accurate anisotropy parameters from the limited structural information available to us). They hence represent an oversimplification but, nonetheless, a useful way to compare the variants. The same trend was observed by comparing ^1^H-^15^N HSQC spectra of the different constructs. Hence, while TPR signals suffer a dramatic line broadening effect in FLΔQ and FL SGTA, the corresponding effect on the N-terminal signals is far less severe (Fig. 6b). These NMR data show that the dynamic parameters of the TPR domain vary depending on whether it is located at one end (N or C) of the protein (TPR, TPR-CTΔQ, NT-TPR constructs) versus being flanked by additional regions on either side (FLΔQ and FL constructs).

Finally, the role of the C-terminal domain in the dimerisation of SGTA was assessed using double electron-electron resonance (DEER) spectroscopy. Four carefully chosen positions were independently labeled along the length of the TPR domain (S88C, S136C, C153, and S197C; Additional file 13: Figure S12) and distances were measured between homologous sites in each monomer in the NT-TPR and FL proteins. Successful labeling was confirmed by continuous-wave (CW) EPR spectroscopy (Additional file 14: Figure S13). The room temperature CW spectra of FL SGTA S88C, C153, and S197C variants reveal relatively mobile spin labels that are thus solvent exposed. In contrast, S136C shows low mobility and is likely constrained by its location inside the TPR groove (Additional file 13: Figure S12). In all cases, deletion of the C-terminus results in slightly greater mobility of the spin labels (Additional file 14: Figure S13) probably due to the contribution of the faster tumbling of the TPR in the absence of the C-terminal domain.

Comparing the DEER traces and corresponding distances for full-length SGTA versus the respective C-terminal deletion mutants (Fig. 6c and Additional file 15: Figure S14), we found that in the full-length protein all four mutants show a similar bimodal distance distribution with a broad set of distances centered around 5 nm and a sharp peak at ca. 7 nm. We conclude that this heterogeneity reflects the presence of flexible linkers that connect the central TPR region to the N- and C-terminal domains of the protein, resulting in a diverse population of conformations.

Deletion of the C-terminal domain has different effects depending on the probe location that is being considered. For S88C, C-terminal deletion has little effect, as expected for a site close to the tight dimerization of the N-terminus, where the relative distances in the dimer would be only affected by different conformations of the linker between the N-terminal and TPR domains which should be similar in both constructs. Moving further away from the N-terminus, deletion of the C-terminal domain is reflected in a lower dipolar frequency and thus longer interspin distances (Fig. 6c). S197C is the probe located furthest from the N-terminus, and in this case, the deletion of the C-terminal domain results in the abolition of almost all short-range interactions and a significant increase of the interspin distance from 7.1 to 7.4 nm. Although efficiently labeled, S197C exhibits shallow modulation depth upon deletion of the C-terminal domain, suggesting that additional longer distances, which are beyond our current resolution, may be present. For the other two labeled positions, we observe intermediate behavior; C153 shows similar results to S88C while the S136C position is more affected by the C-terminal deletion, comparable to S197C (Additional file 15: Figure S14). This is likely due to the positions of both sites relative to the beginning of the TPR. Although further along the primary sequence, in the folded protein, C153 sits closer to the N-terminal end of the TPR domain than S136 does. Thus, like S88C, it is less affected by the deletion of the C-terminal domain (Additional file 13: Figure S12).

**Fig. 7:**
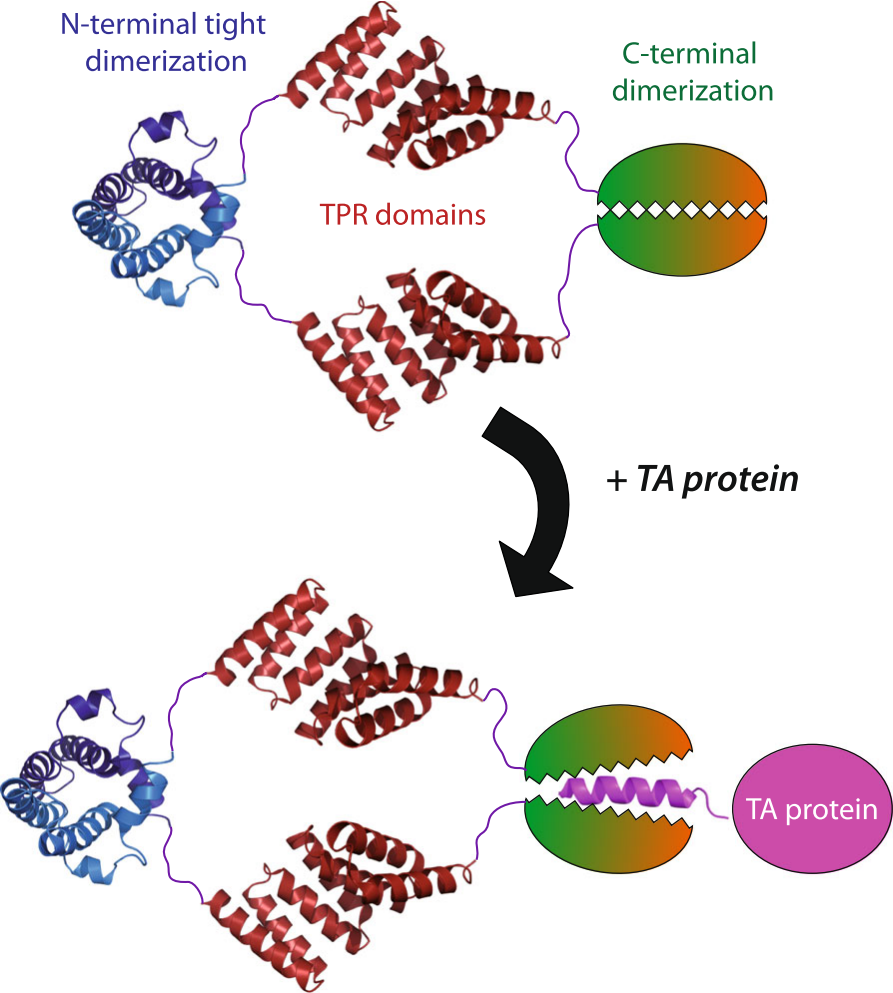
Speculative schematic representation of the hydrophobic substrate recognition of SGTA. SGTA is well known to form a tight dimer through its N-terminal domain. Here, we discovered that the C-terminal region also dimerizes in a more transient or weak fashion, facilitating the capture and/or shielding of hydrophobic substrates such as the transmembrane helices of tail-anchored proteins. The structural arrangement of this C-terminal dimer and its mechanism of hydrophobic substrate binding are still a mystery and constitute an open field for further research

When taken together, these native mass spectrometry, SAXS, NMR relaxation, and EPR data evidence the presence of a constrained conformation of full-length SGTA in solution, where the C-terminal regions of both monomers can interact to close the dimer at this end, thereby bringing the two central TPR domains closer together and constraining their mobility (schematically depicted in Fig. 7).

## Discussion

To date, a complete structural characterization of full-length SGTA remains inaccessible via traditional methods (X-ray crystallography, NMR, cryo-EM), likely due to the presence of flexible linkers between its distinct domains together with the challenging properties of the C-terminal region. Our integrative approach, in which we have combined several structural biology techniques, has now revealed significant insights into the domain organization of SGTA in the context of the full-length protein.

We find that SGTA is a multimodular protein whose three domains are separated by two flexible, non-sequence conserved, linkers thereby leaving these domains independent from each other. The possibility of an independent motion of the TPR relative to the N-terminal domain was first suggested for Sgt2, the fungal homolog of SGTA [24]. Here, we have employed NMR (heteronuclear NOE measurement), SAXS, and EPR, all of which support a similar arrangement for SGTA which extends the model to include C-terminal domain independence.

We have discovered that the C-terminal domain of SGTA is partially structured: it contains one or two α-helical regions, the NNP and likely the Q-rich stretches, but, so far, the intrinsic properties of this domain preclude a typical high-resolution structural study. In addition, our new data reveal that the C-terminal region exists as a dimer in solution as an excised domain (direct detection by native mass spectrometry) and also in the context of the full-length assembly (data from native mass spectrometry, SAXS, NMR relaxation, and EPR experiments).

Whilst the exact nature of this C-terminal domain’s folding and dimerization remains to be fully defined, perturbation of the domain via deletion of the Q-rich region or mutation of the NNP motifs had effects on the structure and/or function of the protein. We have observed in vitro evidence suggesting that both conserved regions are necessary for the correct folding/stability of the domain. The comparison of NMR spectra between CT and CTΔQ constructs shows large chemical shift differences (Additional file 5: Figure S5); moreover, the CTΔQ version is far less stable and more prone to hydrogel formation than the complete C-terminal domain. These facts taken together indicate that the CTΔQ construct is likely to be a structurally impaired protein with a tendency towards aggregation. In addition, mutation of the three NNP stretches affects the capacity of the domain to interact with a putative ligand of ~370 Da detected by mass spectrometry and reduces the stability of the dimer in favor of higher aggregated states (Fig. 3d), although it does not seem to significantly alter the secondary structure (Fig. 3a).

We also find that mutation of the NNP region alters the effect of overexpressing SGTA in a well-established cell culture model [12, 27], leading to the accumulation of a model MLP substrate and its incorporation into cytosolic aggresomes. The likely result of overexpressing a SGTA variant unable to bind hydrophobic substrates would be that steady-state MLP levels are similar to those observed when the PEX19 control is overexpressed, but, on the contrary, we observe a significant increase in OP91 with the 3xNNP/AAA mutant. Hence, the mutation of the NNP region may not abolish the capacity of SGTA to bind to hydrophobic substrates, but rather alter its ability to release the substrate, inhibiting its access to the proteasome and resulting in MLP accumulation. Alternatively, the 3xNNP/AAA mutation may result in a non-functional/unstable aggregation prone form of exogenous SGTA that titrates out endogenous quality control factors and thereby delays the processing and degradation of MLPs. In contrast, although the Q-rich region of SGTA has been implicated in its binding to hydrophobic substrates [30], the deletion of this region does not perceptibly perturb the effects of its overexpression on OP91 when compared to full-length SGTA.

We can only speculate about the structural arrangement that the proposed helical regions may exhibit; the importance of both the NNP and the Q-rich regions for the protein stability indicates that they may participate in the same fold and potentially cooperate to stabilize the hydrophobic substrate. Furthermore, the lack of NMR signals for the two unassigned stretches (one in each of the NNP and Q-rich regions) might also reflect their aggregation tendency and/or their capacity to bind as yet unidentified molecules. We speculate that small hydrophobic compounds or even short peptides (probably the molecules detected by mass spectrometry) may be trapped by the C-terminal region during protein purification thereby creating a heterogeneous sample which results in pronounced NMR chemical exchange in this region. If this is the case, it is surprising that deleting the Q-rich region had no statistically significant effect on steady-state OP91 level in cells, while the same deletion precluded binding of the small molecule as observed by native MS.

Taken together all available data suggest that the NNP region is essential for the correct functioning of the domain, and the Q-rich region is important for the correct folding and stability of the domain and may be involved in assisting substrate recognition.

The ability of SGTA to form a closed dimer conformation has potential implications for its recognition of hydrophobic substrates that will be a key area for future investigation. At present, we can only speculate as to the nature of substrate binding to SGTA. Since we only observe our bound molecule in dimerized C-terminal samples, it is tempting to speculate that an SGTA C-terminal closing action might grab a substrate in a tweezer-like motion, affording all round protection from the aqueous cytoplasm (speculatively depicted in Fig. 7). This would be comparable to the binding of hydrophobic TA proteins by Get3, where a single transmembrane helix binds across the hydrophobic groove of Get3 formed by its dimer interface [19]. The “open” and “closed” forms of Get3, which occur as a function of its nucleotide hydrolysis cycle, have been extensively characterized at a structural level [34]. One of the Get3 helices was suggested to act as a dynamic “lid,” protecting the transmembrane helices from aggregation during the ER delivery process [17, 19]. Since TRC40 (human equivalent of Get3), can receive TA proteins directly from SGTA, the question remains as to how this handover occurs. Unlike SRP54, a component of the signal recognition particle, and TRC40, which both bind comparable hydrophobic substrates, SGTA and BAG6 have no nucleotide binding or hydrolysis capability so their mechanisms for substrate binding and release remain unclear [32].

Nevertheless, the quality control machinery for hydrophobic proteins in the mammalian cytoplasm does appear to rely heavily on multimerisation [6] to link and branch its components. In fact, the SGTA_TPR domain is known to bind to Hsp90 and Hsp70 chaperones, both of which can also form homodimers which undergo conformational changes in conjunction with their nucleotide hydrolysis cycles [35]. In particular, Hsp90, like SGTA, has three domains with a tight dimerization at one end and a transient dimerization at the other, in combination with many accessory proteins that bind along its length [36, 37].

## Conclusions

We have established that SGTA can transiently dimerize at its C-terminal in addition to its tight N-terminal dimerization. This is a potential mechanism for it to surround and protect its hydrophobic substrates (Fig. 7). Our identification of the potential tweezer-like property of SGTA adds important information to our gradually increasing insights into its function and underlines the importance of multimerization-dependent branching to the interactions between factors that mediate intracellular protein quality control.

## Methods

### Cell-based studies

All reagents were from Sigma, unless stated otherwise. Antibodies used were as follows: mouse anti-opsin [18], rabbit anti-V5 (custom made), rabbit anti-V5 (Abcam), and mouse anti-tubulin (gift from Keith Gull). Fluorophore-conjugated secondary antibodies for microscopy and Western blotting were purchased from Molecular Probes and LI-COR Biosciences, respectively. The plasmids for the expression of V5-tagged PEX19, wild-type SGTA, and the TPR mutant were previously described [12]. The 3x NNP/AAA (NNP positions at 226-228, 239-241 and 255-257) and ΔQ (Δ275-313) variants of SGTA-V5 were generated by multisite-directed mutagenesis and inverse PCR, respectively, and validated by DNA sequencing. HeLa cells were cultured in DMEM supplemented with 10% FCS and 2 mM l-glutamine at 37 °C and 5% CO_2_. DNA transfections were performed using Lipofectamine 2000 (Invitrogen) and cells analyzed 20–24 h post-transfection. The inducible HeLa cell line expressing OP91 was generated using the Flp-In T-REx system [12] and maintained in complete DMEM supplemented with 100 μg/ml hygromycin B and 4 μg/ml blasticidin S at alternate passages. At 8 h after DNA transfection, T-REx HeLa cells were treated with DMEM containing 1 μg/ml tetracycline for an additional 12–16 h to induce OP91expression. For Western blot analysis, cells were lysed directly into SDS-PAGE sample buffer. Samples were denatured by 1 h incubation at 37 °C with shaking, and then sonicated 3 × 15 s with the Bioruptor (Diagenode). Proteins were separated by SDS-PAGE and analyzed by infrared immunoblotting. The fluorescent bands were visualized and quantified using Image Studio (LI-COR Biosciences). For immunofluorescence microscopy, cells growing on coverslips were fixed with 3% (*v*/*v*) formaldehyde, permeabilized with 0.1% (*v*/*v*) Triton X-100, and washed with PBS in incubated with primary and secondary antibodies in PBS at room temperature. Coverslips were mounted in ProLong Diamond (Molecular Probes) and analyzed using an Olympus BX60 upright microscope equipped with a MicroMax cooled, slow-scan CCD camera (Roper Scientific) driven by Metaview software (University Imaging Corporation). Images were processed using Adobe Photoshop CS5. Quantification results are expressed as the mean ± s.e.m. from three independent experiments. The statistical significance of the results was assessed by applying a Student’s *t* test using Prism 7 (GraphPad). *P < 0.05, ** *P* < 0.01, *** *P* < 0.001.

### DNA cloning and protein production

SGTA gene fragments encoding the following constructs: NT (1-86, including the linker), TPR (85-213), NT-TPR (1-213), FLΔQ (1-274), TPR-CTΔQ (85-274), CTΔQ (213-274), CT (213-313), and the FL SGTA (1-313) were PCR amplified from cDNA (Life Technologies) and inserted into *Bam*HI/*Xho*I restriction sites of a home-modified pET28 vector, encoding an N-terminal thioredoxin A fusion protein followed by a hexahistidine tag and tobacco etch virus (TEV) protease cleavage site.

Typically, protein expression was carried out in BL21 (DE3) strains after induction with 0.5 mM isopropyl-β-d-thiogalactopyranoside (IPTG) at OD600 ≈ 0.8, followed by overnight incubation at 20 °C. For labeled proteins, growth was carried out in M9 media supplemented with labeled ammonium chloride (> 98% ^15^N, Sigma-Aldrich), glucose (> 99% U-^13^C, Sigma-Aldrich), and/or 100% D_2_O (Sigma-Aldrich).

Harvested cells were resuspended in lysis buffer (20 mM potassium phosphate, pH 8.0, 300 mM NaCl, 10 mM Imidazole, 250 μM TCEP), supplemented with protease inhibitors (0.3 μM Aprotinin, 10 μM Leupeptin, and 1 μM Pepstatin A), and 1 mM PMSF and lysed by sonication or cell disruptor (Constant Systems Ltd.). Cell debris was removed by centrifugation, and the soluble fractions were purified using nickel affinity chromatography (HisTrapTM HP 5 ml, GE Healthcare) and eluted with buffer containing 300 mM imidazole, followed by dialysis into cleavage buffer (20 mM potassium phosphate, pH 8.0 and 300 mM NaCl) and digestion with homemade TEV protease (≈ 100 μg/ml) at 4 °C overnight. After removing the fusion protein and the histidine tag by nickel affinity chromatography, the target protein was recovered in the flow through and gel filtration steps were carried out using HiLoad 16/60 Superdex 200 column (GE Healthcare), previously equilibrated in buffer containing 10 mM potassium phosphate pH 6.0, 100 mM NaCl and 250 μM TCEP. Proteins were concentrated using Vivaspin concentrators (Sartorius Stedin) and the sample purity and homogeneity was assessed by SDS-PAGE and NMR.

### Biochemical characterization

Analytical size-exclusion chromatography (SEC) was performed using Superdex 200 10/300 GL column (GE Healthcare) pre-equilibrated with 10 mM KPi, 100 mM NaCl, pH 6.0 buffer. Molecular mass was estimated based on the migration of protein standards on the SEC column (aprotinin – 6.5 kDa, ribonuclease A – 13.7 kDa, carbonic anhydrase – 29.0 kDa, ovalbumin – 44.0 kDa, conalbumin – 75.0 kDa, and aldolase – 158.0 kDa; GE).

Circular dichroism (CD) spectra of CT, CT 3x NNP/AAA and CTΔQ SGTA constructs were acquired using an Aviv Circular Dichroism Spectrophotometer, Model 410 (Biomedical Inc., Lakewood, NJ, USA). Protein samples were adjusted to 0.5 mg/ml in 10 mM KPi, 100 mM NaCl, and pH 6.0 buffer, and the experiments were recorded using a rectangular demountable Suprasil quartz cell of 0.1 mm pathlength (Hellma Analytics). Each sample was scanned three times from 260 to 195 nm, at 1-nm intervals with an averaging time of 0.5 s. After the background subtraction for all CD spectra, data were converted to mean residue molar ellipticity and deconvoluted using SELCON3 [38].

Dynamic light scattering (DLS) was performed using a Nanosizer S diffraction particle sizer (Malvern Instruments, UK) with a 5003 multi-digital correlator. The light source was a 2 mW He-Ne laser, linearly polarized, with *λ* = 633 nm, and scattering angle *θ* = 173°. Samples were prepared at 0.5 mg/ml in 10 mM KPi, 100 mM NaCl, and pH 6.0 buffer and loaded into 0.5 ml volume disposable cuvettes (Sigma, Poole, UK). The experiments were measured at room temperature in triplicate.

### NMR

For NMR experiments, protein samples were prepared at concentrations between 200 and 500 μM containing 10% D_2_O (Sigma-Aldrich) in 10 mM potassium phosphate pH 6.0, 100 mM NaCl, and 250 μM TCEP buffer (supplemented with 10 μM DSS for proton chemical shift referencing). All experiments were acquired in 5 mm NMR tubes at 25 °C using Bruker Avance spectrometers at 500, 700, or 950 MHz equipped with cryoprobes and controlled by the TopSpin 3.1 software package. Backbone assignments were carried out using 3D experiments (HNCO, HN(CA)CO, CBCA(CO)NH, and CBCANH) [39] for CT, TPR-CTΔQ, and NT constructs (respective BMRB accession numbers: 27272, 27275, and 27276); assignment of the other constructs was compiled using the same information. NMRPipe [40] and CcpNMR Analysis [41] were used for spectral processing and analysis.

#### Optimization

Since two stretches of peaks were missing from our SGTA_CT spectra, we tested numerous conditions to optimize the experiments and potentially reveal the missing peaks. We produced a variety of different SGTA_CT constructs, removing potentially aggregating regions (CTΔQ) and adding the contiguous stable domain, TPR (TPR_CT and TPR_CTΔQ). For each of these, we ran ^1^H-^15^N HSQC experiments at a range of temperatures (Additional file 4: Figure S4), pH (6.0–8.9), protein concentrations (10–500 μM), and with the addition of detergents at various concentrations (DDM or OG at 0.05–0.2%).

#### Relaxation

NMR relaxation experiments were performed for NT, TPR, NT-TPR, TPR-CTΔQ, and FL constructs at concentrations between 200 and 400 μM. ^15^N - {^1^H} heteronuclear measurements were obtained from the ratio of crosspeak volumes between two experiments recorded with 4 s of interscan delay (equilibrium) or 4 s of proton saturation (saturated). A spectrum series with 30.8, 61.6, 123.2, 246.4, 369.6, 554.4, 739.2, 985.5, 1232, 1386, and 1540 ms of inversion-and-recovery delays and 16.96, 33.92, 50.88, 67.84, 84.8, 118.72, 152.64, 186.56, 220.48, and 254.4 ms of CPMG echo delays was recorded for *T*_1_ and *T*_2_ measurements, respectively. ^15^N *T*_1_ and *T*_2_ relaxation times were computed using standard methods analogous to previous approach [42], from the single exponential decay fitting of the peak intensities for each amide signal. Correlation times (*τ*_c_) have been estimated from the *T*_1_/*T*_2_ averaged values for each domain (NT comprising residues from 5 to 65 and TPR from 87 to 206) using the following equation:

$$ {\tau}_c\approx \frac{1}{4\pi {\upsilon}_N}\sqrt{6\frac{T_1}{T_2}-7} $$

Values for *n* were as follows: NT = residues 5–65: in construct NT, *n* = 57; in construct NT-TPR, *n* = 45; and in construct FL, *n* = 37 and TPR = residues 87–205: in construct TPR, *n* = 97; in construct NT-TPR, *n* = 81; in construct TPR-CTΔQ, *n* = 92; and in construct FL *n* = 40.

### Native mass spectrometry

Mass spectra of SGTA samples (NT-TPR, FLΔQ, FL, CT, and CT 3x NNP/AAA mutant) were recorded on a Synapt HD mass spectrometer (Waters) modified for studying high masses. Protein samples were exchanged into 0.20–0.75 M ammonium acetate (pH 7.0) solution using Micro Bio–Spin 6 chromatography columns (Bio-Rad) and diluted to a final concentration of 5–10 μM before analysis. 2.5 μL of protein solution was electrosprayed from a borosilicate emitter (Thermo Scientific) for sampling. Typical conditions were capillary voltage 1.8–2.5 kV, cone voltage 60–120 V, collision voltage 10–30 V, with backing pressure 3–4 mbar, and source temperature of 20 °C. Spectra were calibrated externally using cesium iodide. Data acquisition and processing were performed using MassLynx 4.1.

### SAXS

Small-angle X-ray scattering data were collected at the EMBL beamline P12 at PETRA 3 storage ring (DESY, Hamburg). All measurements were carried in 10 mM KPi, 100 mM NaCl, pH 6.0, buffer at 25 °C with protein solutions at concentrations ranging from 0.5 to 8.8 mg/ml (for NT-TPR and FL SGTA). The experiments were recorded using a PILATUS 2-M detector (DECTRIS, Switzerland) at a sample-detector distance of 3.1 m and a wavelength of *λ* = 0.12 nm, covering the range of momentum transfer 0.07 < s < 4.80 nm^−1^ (*s* = 4π sinθ/λ, where 2θ is the scattering angle). The measurements were taken in an in-vacuum capillary; no measurable radiation damage was detected by comparison of 20 successive frames with 50-ms exposures. The experimental scattering profiles from all solutes were corrected for the solvent scattering, normalized against transmitted intensity and sample concentration, and processed using standard protocols [43]. Extrapolation to infinite dilution and merging of different data sets were performed with PRIMUS [43]. The overall parameters such as radius of gyration (*R*_g_), the maximum particle dimension (*D*_max_), and the Porod volume (*V*_p_) were evaluated using standard procedures [43]. The program GNOM [44] was used to calculate the distance distribution function. The molecular weight (MW) was estimated by comparing the forward scattering with that of a standard protein (bovine serum albumin).

The flexibility of the different constructs was compared using the program EOM 2.0 [45]: EOM 2.0 is a program that fits the averaged theoretical scattering intensity from an ensemble of conformations into the experimental SAXS data. A pool of *n*-independent models based upon sequence and structural information was first generated. Then, a genetic algorithm was performed for the selection of the ensemble of conformations that best fit the data. High-resolution structures for individual subunits, if available, were used as constraints for the generation of the pool. Data were deposited in the SASBDB [46] under accession codes: SASDDB6 and SASDDC6.

### TPR mutant design, spin labelling, and EPR sample preparation

SGTA contains four cysteine residues (C38, C129, C148, and C153), and to prepare the single cysteine mutants in NT-TPR and FL SGTA constructs we first changed all wild-type cysteine amino acids into serine residues using site-directed mutagenesis. We confirmed that the folding of the resultant cysteine-free protein is conserved using NMR. Then, four positions were selected (S88, S136, C153, and S197) to contain the solvent-exposed cysteine for spin labeling. These different mutants were created as well using site-directed mutagenesis (except in the case of the wild-type C153). Proteins were prepared in the same way as the wild-type versions.

Two milliliters of 0.25 mM SGTA (NT-TRP and FL mutants) in 20 mM potassium phosphate and 300 mM NaCl buffer at pH 8.0 were incubated with 150 μl of 37.8 mM (1-oxyl-2,2,5,5-tetramethylpyrroline-3-methyl) methanethiosulfonate (MTSL, Santa Cruz Biotechnology) spin label (~ 20-fold excess) overnight at 4 °C in the dark. The spin label was removed by size-exclusion chromatography using HiLoad 16/60 Superdex 200 column (GE Healthcare), previously equilibrated in buffer containing 20 mM potassium phosphate pH 7.0 and 100 mM NaCl. Continuous-wave EPR spectra were acquired on samples in the elution buffer, whereas PELDOR samples were exchanged into D_2_O-containing buffer (Sigma-Aldrich) before dilution with 50% d8-glycerol (Sigma-Aldrich). The elution volume of each labeled mutant following size-exclusion chromatography was similar for all labeled proteins, indicating similar stability of the mutants and the wild-type SGTA. To make EPR samples (final concentration 300–500 μM), ~ 200 μL of a given sample were transferred to a borosilicate glass tube (O.D. 5 mm, Wilmad 500 MHz precision). Labeling efficiencies were above 70%, as determined by continuous-wave EPR [47].

### EPR spectroscopy

Continuous-wave EPR measurements were conducted at room temperature on a Bruker *E-scan* bench top spectrometer, with 1-mW microwave power, 0.1-mT modulation amplitude and 20-ms conversion time. After flash freezing the samples in liquid nitrogen, DEER measurements were performed at 50 K on an ELEXSYS E500 spectrometer (Bruker) operating at 9.6 GHz equipped with an ER 4118 X-MD5 resonator, a cryogen-free close-circuit cryostat (Cryogenics Ltd.) and a Lakeshore temperature controller. The four-pulse double electron-electron resonance sequence [48] used was π/2(*ν*_*obs*_)-*τ*_l_-π(ν_*obs*_)-*t*-π(ν_*pump*_)-(*τ*_l_ + *τ*_2_-*t*)-π(*ν*_*obs*_)-*τ*_*2*_*-echo*, where the observer pulse lengths were 16 and 32 ns for the π/2 and π pulses, respectively. The pump pulse length (π(*ν*_*pump*_)) was 12 ns and *τ*_2_ was 7 or 8 μs. All other parameters, namely *τ*_1_ = 400 ns and Δ*τ*_1_ = 56 ns for nuclear modulation averaging, were selected as described earlier [48]. Data points were collected in 16 ns time steps. The acquisition time for each DEER spectrum was between 3 and 12 h. Time-domain spectra were analyzed using the program DeerAnalysis2016 [49]. A homogeneous three-dimensional fit was used as background correction and the distance distributions computed by Tikhonov regularization.

## Additional files

Additional file 1: **Figure S1.** Sequence alignment of human SGTA (*Homo sapiens*) and several homolog proteins: Sumatran orangutan (*Pongo abelii*), white-cheeked gibbon (*Nomascus leucogenys*), dog (*Cannis lupus familiaris*), pig (*Sus scrofa*), rat (*Rattus norvegicus*), chicken (*Gallus gallus*), African clawed frog (*Xenopus laevis*), gray short-tailed opossum (*Monodelphis domestica*), three-spined stickleback (*Gasterosteus aculeatus*), Japanese pufferfish (*Takifugu rubripes*), American chameleon (*Anolis carolinensis*), Spotted green pufferfish (*Tetraodon nigroviridis*), Japanese rice fish (*Oryzias latipes*), and West Indian ocean coelacanth (*Latimeria chalumnae*). All proteins were selected using the BLAST tool with the hsSGTA sequence as query; the alignment was obtained using Jalview 2.7. (PDF 1115 kb)

Additional file 2: **Figure S2.** The SGTA-3xNNP/AAA-V5 mutant stimulates the accumulation of OP91 in discrete cytosolic inclusions. HeLa cells stably expressing OP91 under an inducible promoter were transiently transfected with either a control plasmid (PEX19-V5) or plasmids encoding V5-tagged SGTA variants as indicated, and then induced to express OP91. Cells were fixed, stained for opsin and the V5 epitope, and analyzed by fluorescence microscopy. Opsin-positive inclusions in SGTA-3xNNP/AAA-V5-expressing cells are indicated by arrows. Scale bar is 10 μm. (PDF 19499 kb)

Additional file 3: **Figure S3.** SGTA-3xNNP/AAA-V5-expressing cells form cytosolic inclusions in the absence of an MLP substrate. HeLa cells stably expressing OP91 under an inducible promoter were transiently transfected with plasmids encoding full-length or 3xNNP/AAA SGTA-V5. Uninduced cells were fixed, stained for opsin and the V5 epitope, and analyzed by fluorescence microscopy. V5-positive cytosolic inclusions were observed exclusively in SGTA-3xNNP/AAA-V5 expressing cells. Scale bar is 10 μm. (PDF 6818 kb)

Additional file 4: **Figure S4.** Overlaid ^1^H-^15^N HSQC spectra of TPR-CT∆Q SGTA at a range of temperatures from 5 °C (gray-blue) to 40 °C (maroon). (PDF 3684 kb)

Additional file 5: **Figure S5.** Complete analysis of the chemical shift difference between SGTA constructs for the same backbone amide signal in ^1^H-^15^N HSQC spectra. N-terminal, TPR, C-terminal, and other comparisons appear in consecutive pages. (PDF 3505 kb)

Additional file 6: **Figure S6.** Chemical shift index analysis of full-length SGTA, showing the alpha carbon and carbonyl chemical shift deviation from random coil values. (PDF 571 kb)

Additional file 7: **Figure S7.** (A) Size-exclusion chromatography of some different variants of SGTA. Note the unexpected elution volume of the CT construct (red). (B) Dynamic light scattering intensity distributions for CT and CTΔQ constructs showing the size of the most abundant species (~ 9 nm diameter) and some aggregates (more populated in the CTΔQ version). (C) Size-exclusion chromatography of the C-terminal variants. The column utilized for panel C is different from panel A and the calibration varies by ~ 1 ml. (PDF 80 kb)

Additional file 8: **Figure S8.** Mass spectrometry analysis of SGTA CT construct, showing that the protein is not covalently modified. Expected molecular weight = 10,877 Da. (PDF 2643 kb)

Additional file 9: **Figure S9.** Overlaid ^1^H-^15^N HSQC spectra of different SGTA constructs under the same conditions. (A) SGTA FL (black), NT (blue), TPR (red), and CT (green) proteins. (B) SGTA NT-TPR (black), NT (blue), and TPR (red) constructs. (C) SGTA TPR-CTΔQ (black), TPR (red), and CTΔQ (green) versions. (D) SGTA CT (black) and CTΔQ constructs (green). (PDF 271 kb)

Additional file 10: **Figure S10.** ^15^N - {^1^H} heteronuclear NOE measurements of different SGTA constructs; in all cases, the domain boundaries and linker regions are similar. (PDF 142 kb)

Additional file 11: **Table S1.** Detailed SAXS data collection and derived parameters for FL and NT-TPR constructs of SGTA. (DOCX 46 kb)

Additional file 12: **Figure S11.** ^15^N NMR relaxation analysis of N-terminal (A) and TPR (B) domains in different SGTA constructs. NT = residues 5–65: in construct NT, *n* = 57; in construct NT-TPR, *n* = 45; and in construct FL, *n* = 37. TPR = residues 87–205: in construct TPR, *n* = 97; in construct NT-TPR, *n* = 81; in construct TPR-CTΔQ, *n* = 92; and in construct FL *n* = 40. Boxplots show the *T*_1_ and *T*_2_ values obtained for residues of each domain presenting the median, the interquartile range (colored boxes), the maximum and minimum values (segments with whiskers), and the outliers (dots); the correlation times (shown above) were calculated using the averaged value and the standard deviation as set in the “Methods” section. (PDF 4187 kb)

Additional file 13: **Figure S12.** Selected mutations in the TPR domain for MTSL labelling in the EPR experiments. (A) Cartoon representation of the TPR domain with the four positions depicted as green balls. (B) Surface representation of the TPR domain with the four residues colored in green. Notice that the S136C mutation is inside the TPR groove. (PDF 129 kb)

Additional file 14: **Figure S13.** CW-EPR spectra of the FL and C-terminal deleted (NT-TPR) SGTA proteins. Room Temperature CW-EPR spectra for SGTA FL mutants (blue lines) and corresponding NT-TPR constructs (red lines). CW-EPR spectra mainly provide information about the mobility of the spin labels and thus about the local environment of the labeled residues. Spectra of the FL protein are slightly broader than those corresponding to the C-terminal deletion (NT-TPR), as highlighted by arrows in the figure, suggesting that the absence C-terminal domain increase the overall mobility of the TPR domain. (PDF 559 kb)

Additional file 15: **Figure S14.** DEER measurements and distances determined for FL and NT-TPR SGTA constructs spin-labeled in the four SGTA mutants. (i) Primary frequency-domain DEER data. (ii) Background-corrected dipolar evolution data (black lines) and corresponding fits obtained through DeerAnalysis2016 [49] by Tikhonov regularization. (iii) Distance distributions obtained by Tikhonov regularization. Blue fits: FL SGTA; red fits: NT-TPR construct. (PDF 3125 kb)
