# Supplementary material for: Structural complexity of the co-chaperone SGTA: a conserved C-terminal region is implicated in dimerization and substrate quality control
Source: BMC Biol. 2018 Jul 11;16:76. doi: 10.1186/s12915-018-0542-3 (PMC6042327; doi:10.1186/s12915-018-0542-3)
Supplement: Supplementary file 1 — Figure S1. Sequence alignment of human SGTA (Homo sapiens) and several homolog proteins: Sumatran orangutan (Pongo abelii), white-cheeked gibbon (Nomascus leucogenys), dog (Cannis lupus familiaris), pig (Sus scrofa), rat (Rattus norvegicus), chicken (Gallus gallus), African clawed frog (Xenopus laevis), gray short-tailed opossum (Monodelphis domestica), three-spined stickleback (Gasterosteus aculeatus), Japanese pufferfish (Takifugu rubripes), American chameleon (Anolis carolinensis), Spotted green pufferfish (Tetraodon nigroviridis), Japanese rice fish (Oryzias latipes), and West Indian ocean coelacanth (Latimeria chalumnae). All proteins were selected using the BLAST tool with the hsSGTA sequence as query; the alignment was obtained using Jalview 2.7. (PDF 1115 kb) [file 12915_2018_542_MOESM1_ESM.pdf]

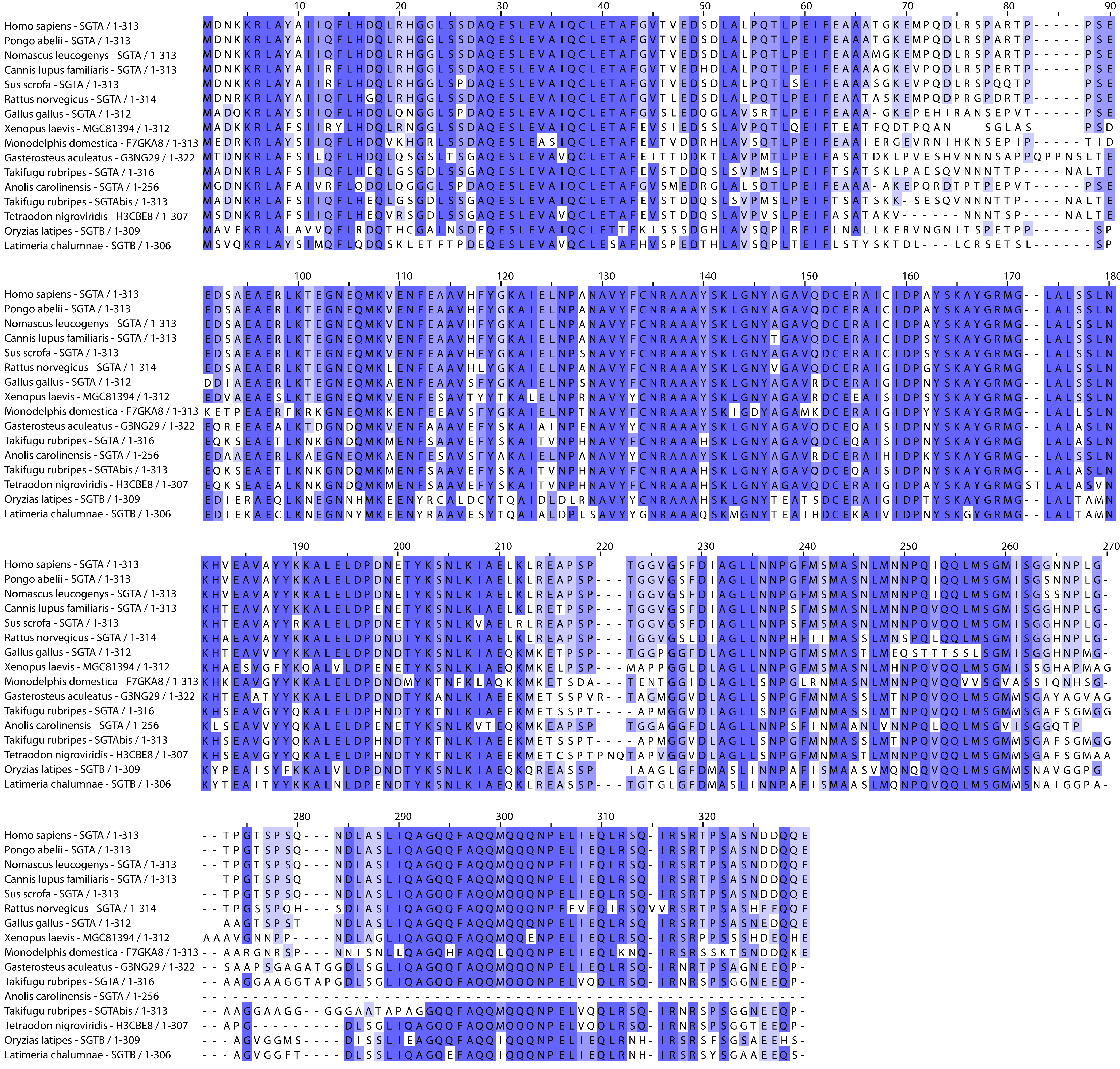

10 20 30 40 50 60 70 80 90
100 110 120 130 140 150 160 170 180
190 200 210 220 230 240 250 260 270
280 290 300 310 320
Homo sapiens - SGTA / 1-313
Pongo abelii - SGTA / 1-313
Nomascus leucogenys - SGTA / 1-313
Cannis lupus familiaris - SGTA / 1-313
Sus scrofa - SGTA / 1-313
Rattus norvegicus - SGTA / 1-314
Gallus gallus - SGTA / 1-312
Xenopus laevis - MGC81394 / 1-312
Monodelphis domestica - F7GKA8 / 1-313
Gasterosteus aculeatus - G3NG29 / 1-322
Takifugu rubripes - SGTA / 1-316
Anolis carolinensis - SGTA / 1-256
Takifugu rubripes - SGTAbis / 1-313
Tetraodon nigroviridis - H3CBE8 / 1-307
Oryzias latipes - SGTB / 1-309
Latimeria chalumnae - SGTB / 1-306
