# Supplementary figures and images for: Structural complexity of the co-chaperone SGTA: a conserved C-terminal region is implicated in dimerization and substrate quality control
Source: BMC Biol. 2018 Jul 11;16:76. doi: 10.1186/s12915-018-0542-3 (PMC6042327; doi:10.1186/s12915-018-0542-3)

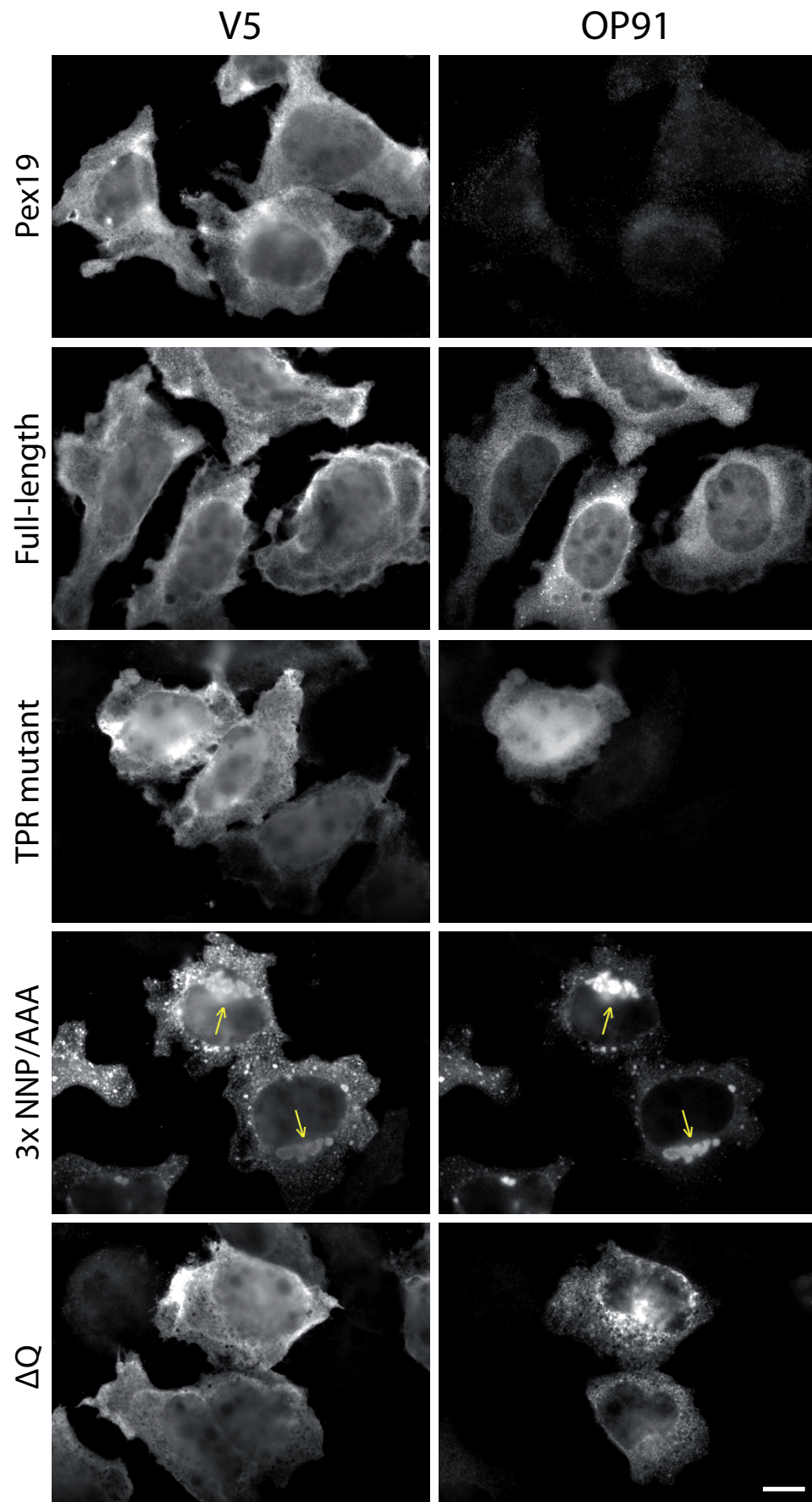

Supplement: Supplementary file 2 — Figure S2. The SGTA-3xNNP/AAA-V5 mutant stimulates the accumulation of OP91 in discrete cytosolic inclusions. HeLa cells stably expressing OP91 under an inducible promoter were transiently transfected with either a control plasmid (PEX19-V5) or plasmids encoding V5-tagged SGTA variants as indicated, and then induced to express OP91. Cells were fixed, stained for opsin and the V5 epitope, and analyzed by fluorescence microscopy. Opsin-positive inclusions in SGTA-3xNNP/AAA-V5-expressing cells are indicated by arrows. Scale bar is 10 μm. (PDF 19499 kb) [file 12915_2018_542_MOESM2_ESM.pdf]

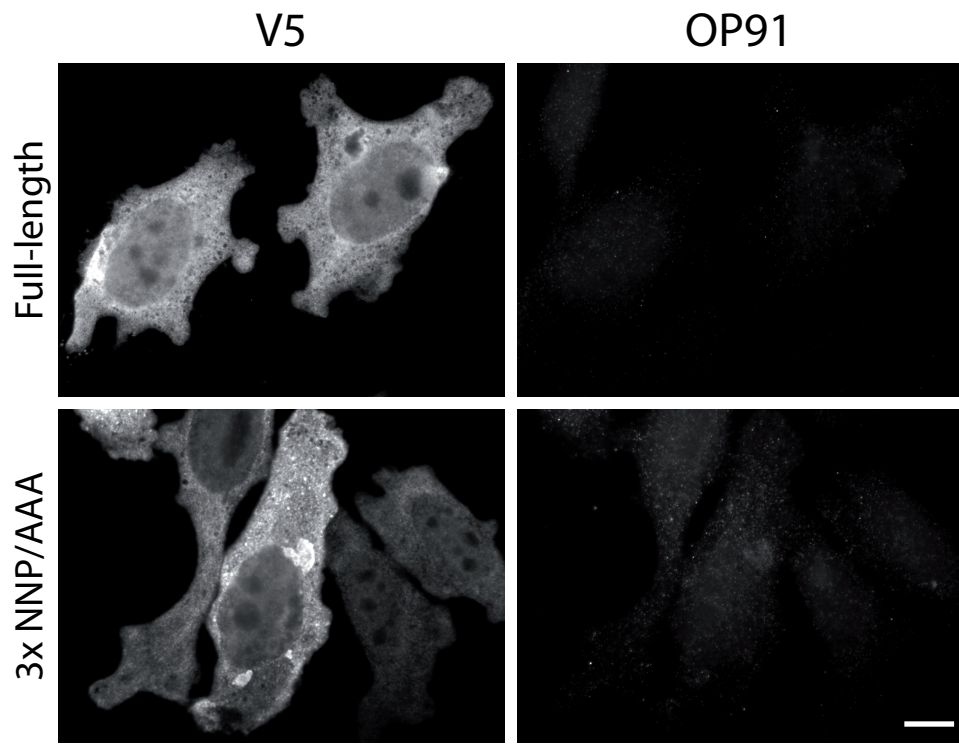

Supplement: Supplementary file 3 — Figure S3. SGTA-3xNNP/AAA-V5-expressing cells form cytosolic inclusions in the absence of an MLP substrate. HeLa cells stably expressing OP91 under an inducible promoter were transiently transfected with plasmids encoding full-length or 3xNNP/AAA SGTA-V5. Uninduced cells were fixed, stained for opsin and the V5 epitope, and analyzed by fluorescence microscopy. V5-positive cytosolic inclusions were observed exclusively in SGTA-3xNNP/AAA-V5 expressing cells. Scale bar is 10 μm. (PDF 6818 kb) [file 12915_2018_542_MOESM3_ESM.pdf]

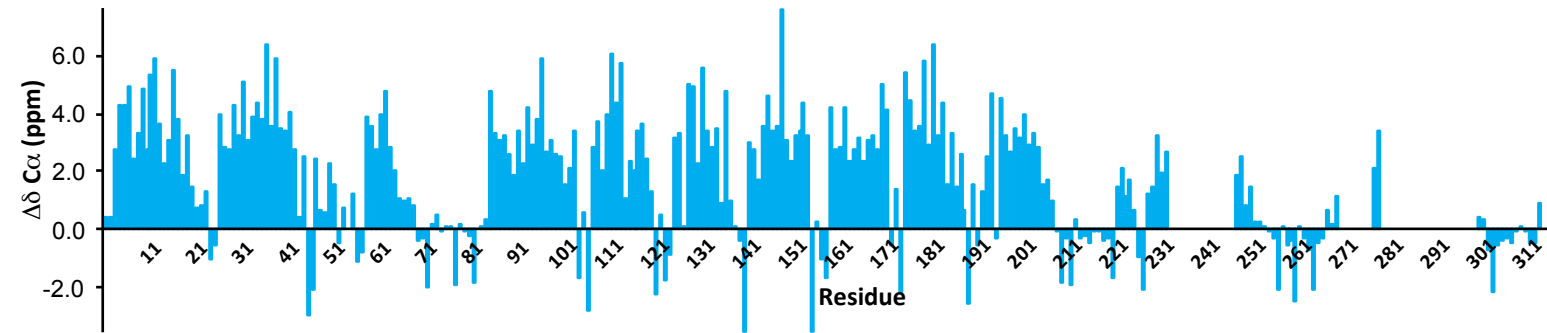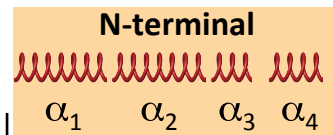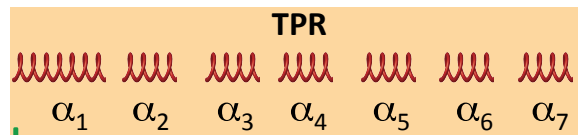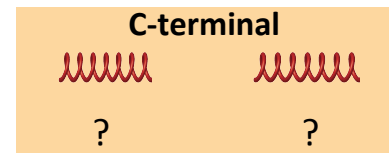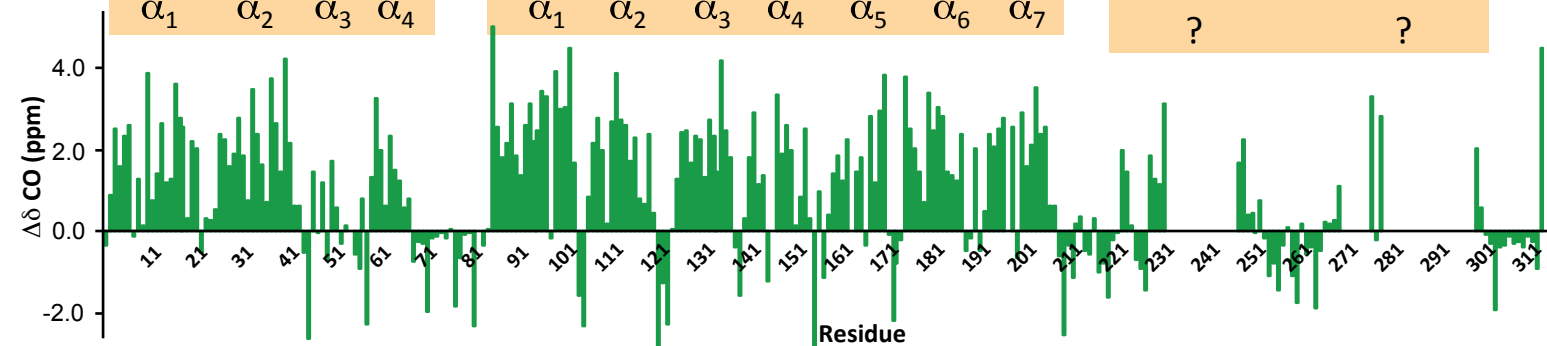

Supplement: Supplementary file 5 — Figure S5. Complete analysis of the chemical shift difference between SGTA constructs for the same backbone amide signal in 1H-15N HSQC spectra. N-terminal, TPR, C-terminal, and other comparisons appear in consecutive pages. (PDF 3505 kb) [file 12915_2018_542_MOESM5_ESM.pdf]

**A**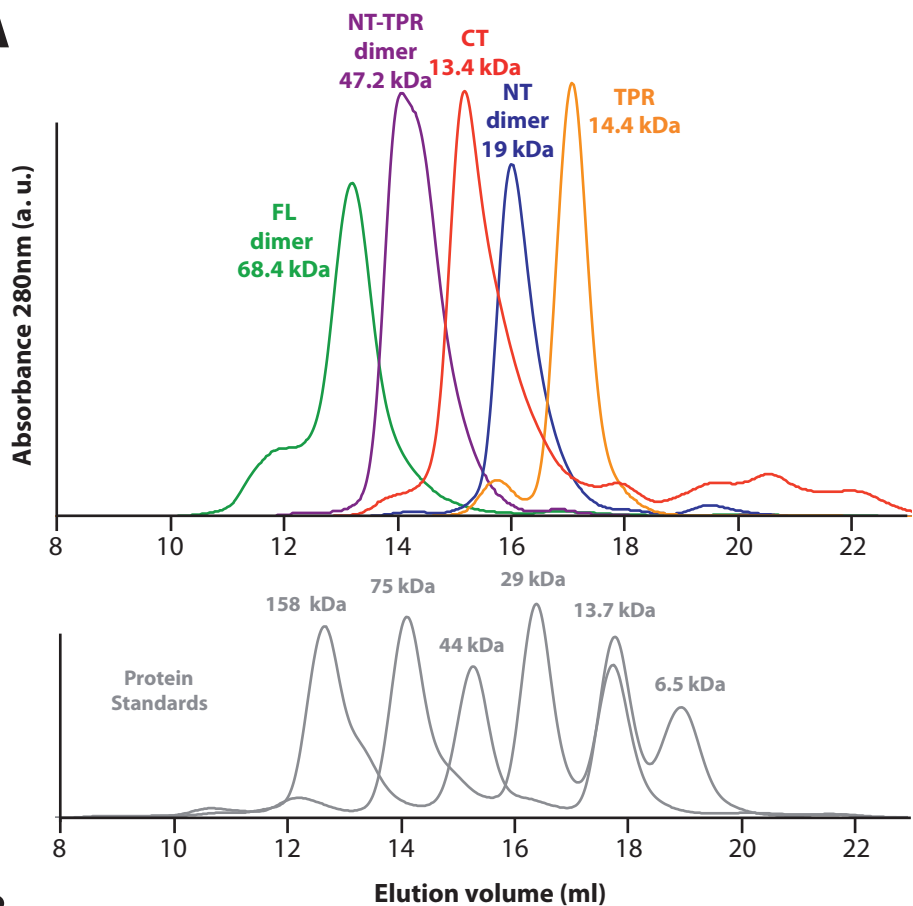**B**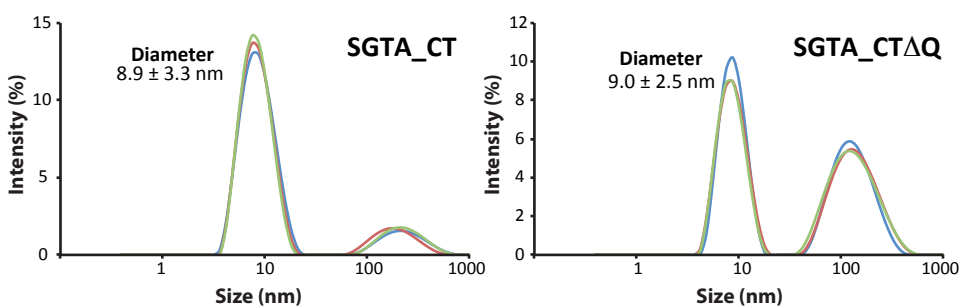**C**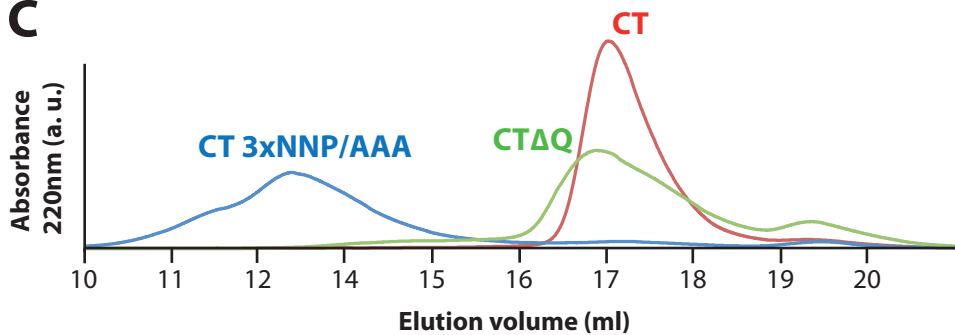

Supplement: Supplementary file 6 — Figure S6. Chemical shift index analysis of full-length SGTA, showing the alpha carbon and carbonyl chemical shift deviation from random coil values. (PDF 571 kb) [file 12915_2018_542_MOESM6_ESM.pdf]

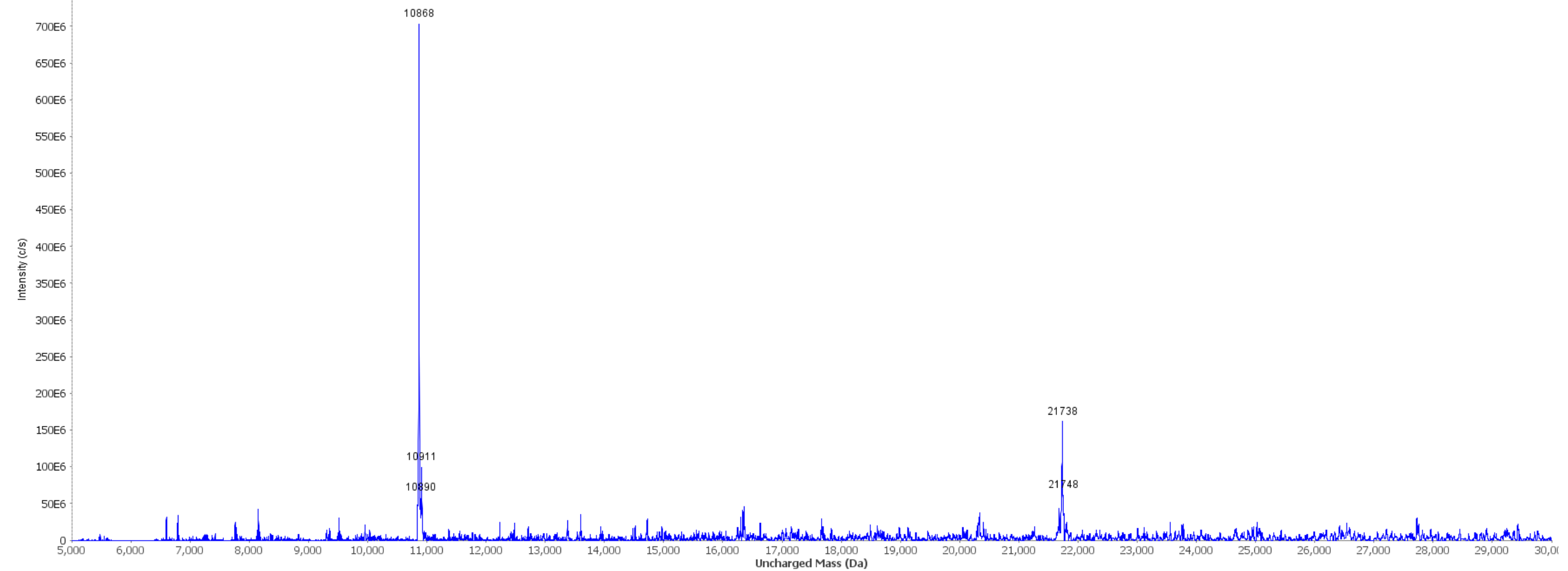

Supplement: Supplementary file 7 — Figure S7. (A) Size-exclusion chromatography of some different variants of SGTA. Note the unexpected elution volume of the CT construct (red). (B) Dynamic light scattering intensity distributions for CT and CTΔQ constructs showing the size of the most abundant species (~ 9 nm diameter) and some aggregates (more populated in the CTΔQ version). (C) Size-exclusion chromatography of the C-terminal variants. The column utilized for panel C is different from panel A and the calibration varies by ~ 1 ml. (PDF 80 kb) [file 12915_2018_542_MOESM7_ESM.pdf]

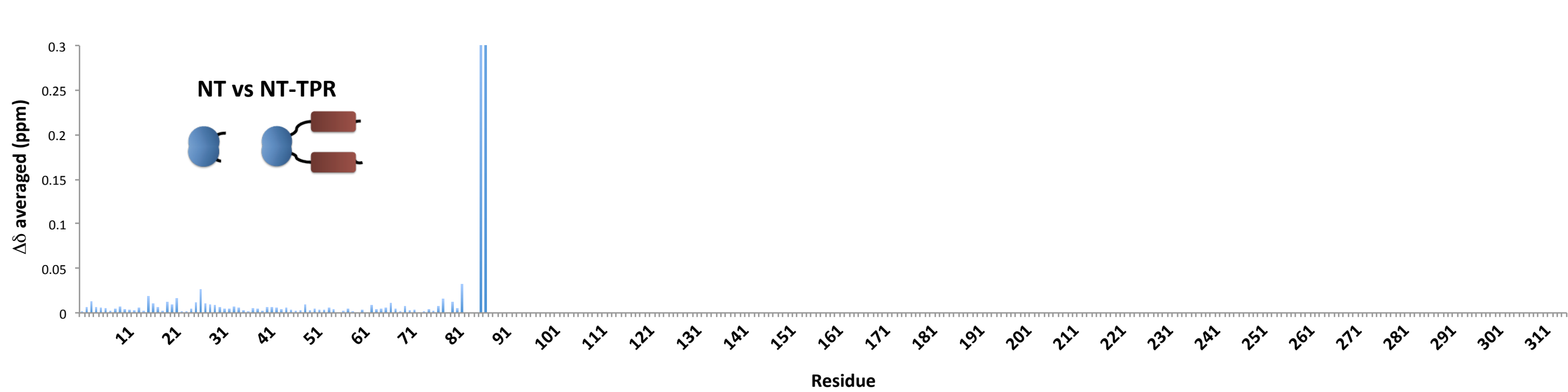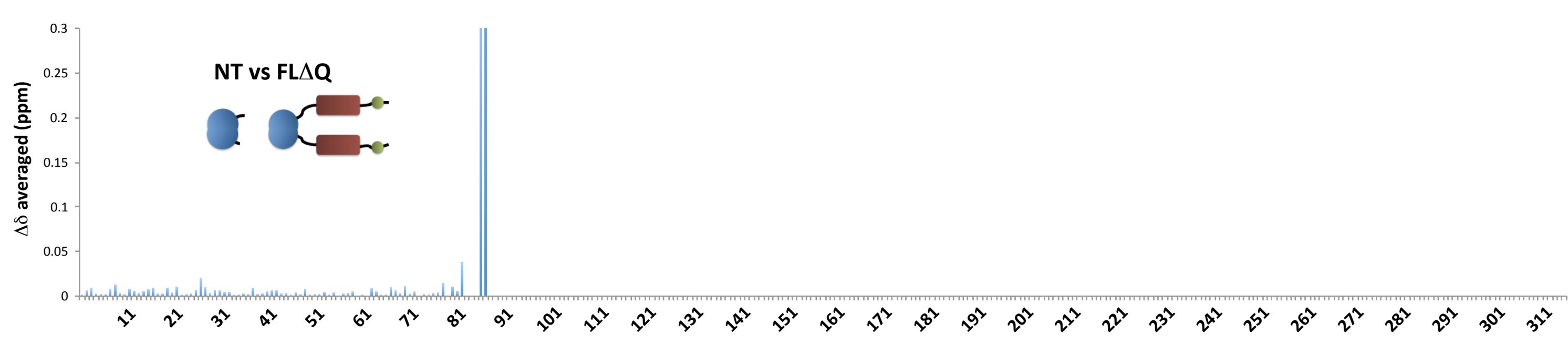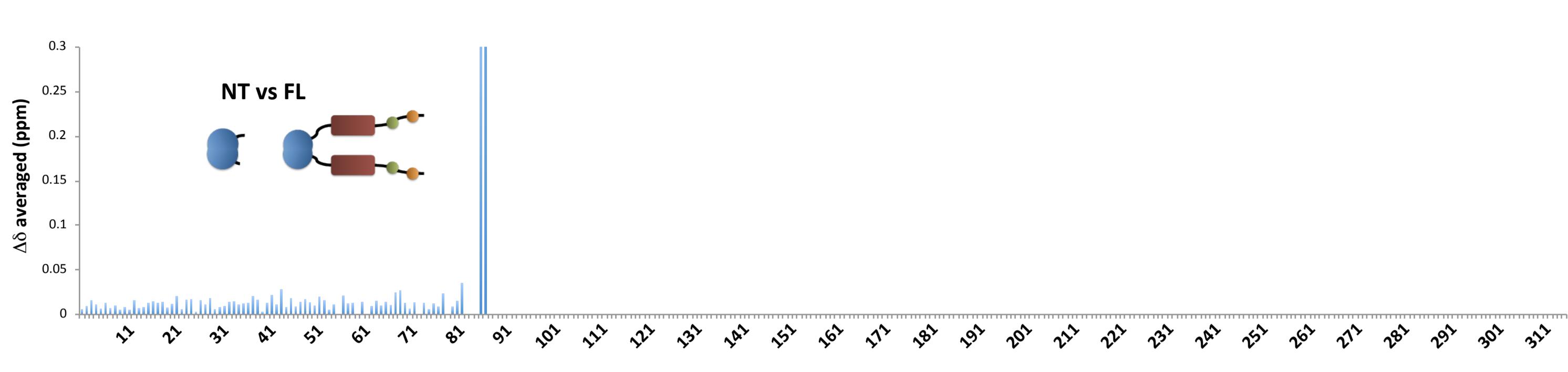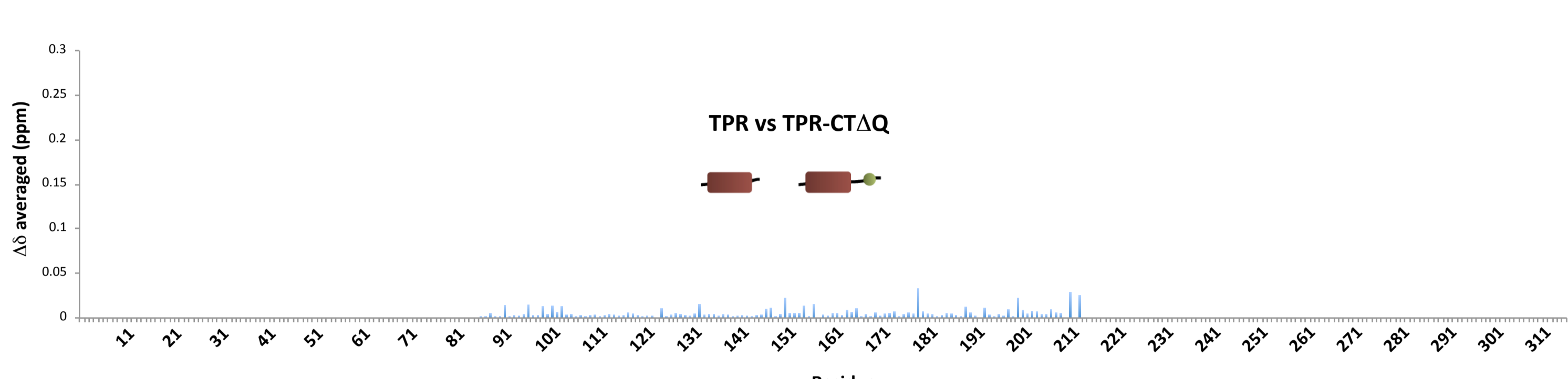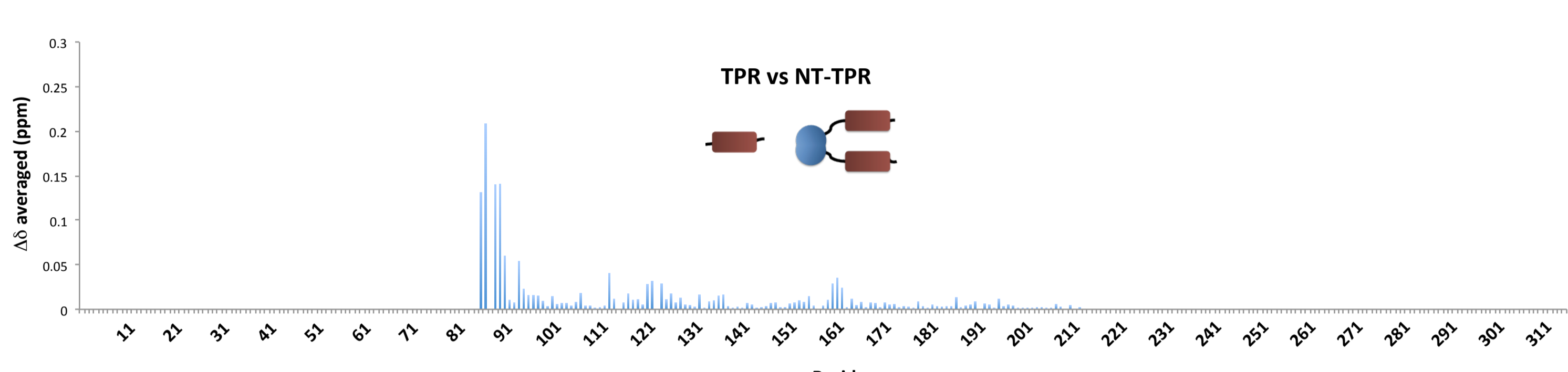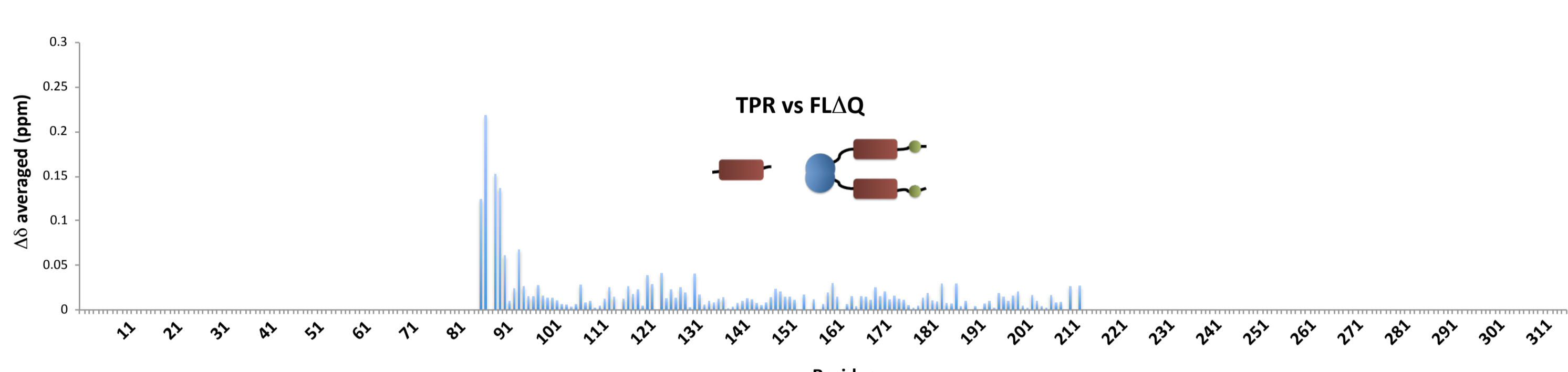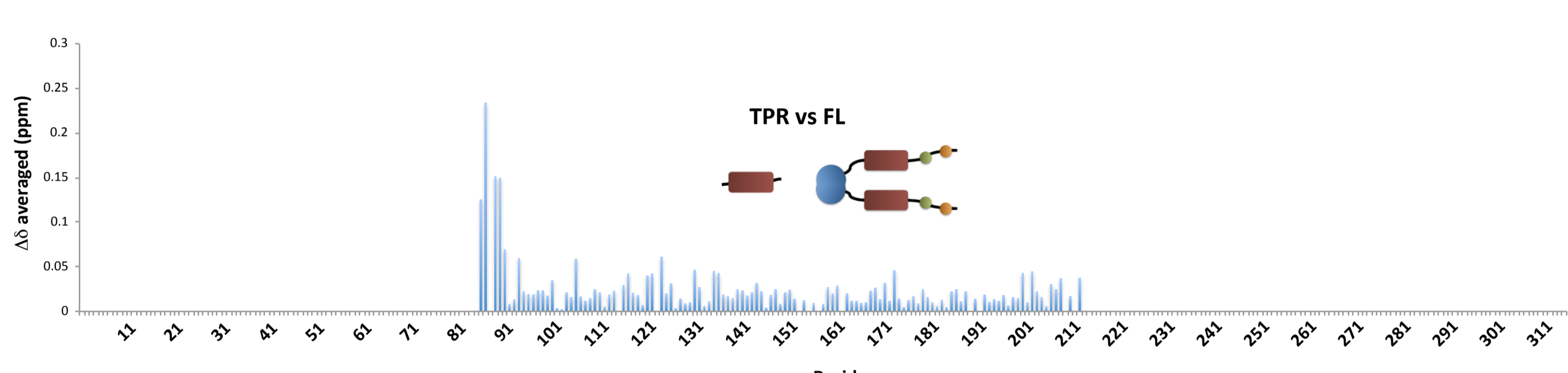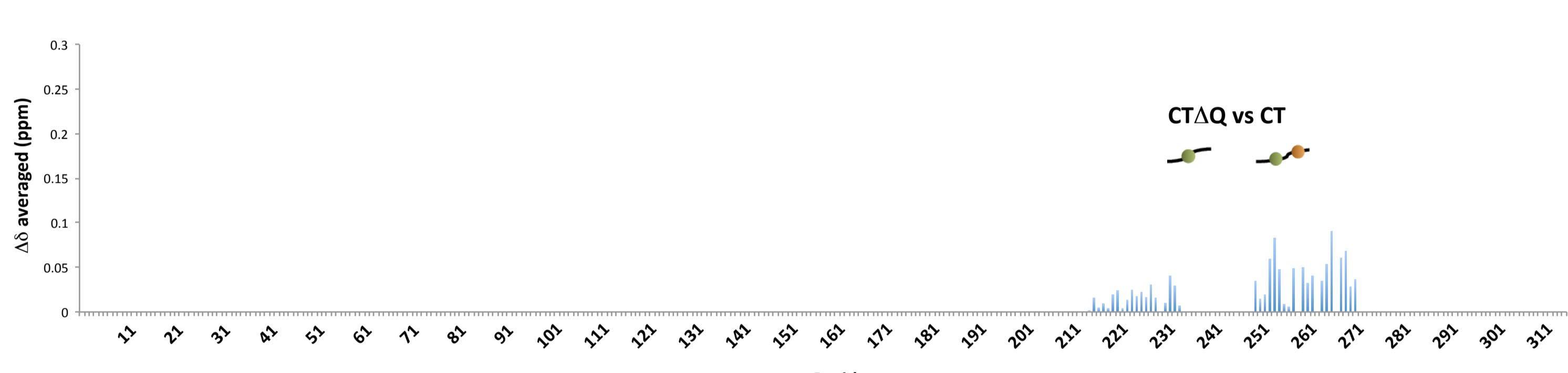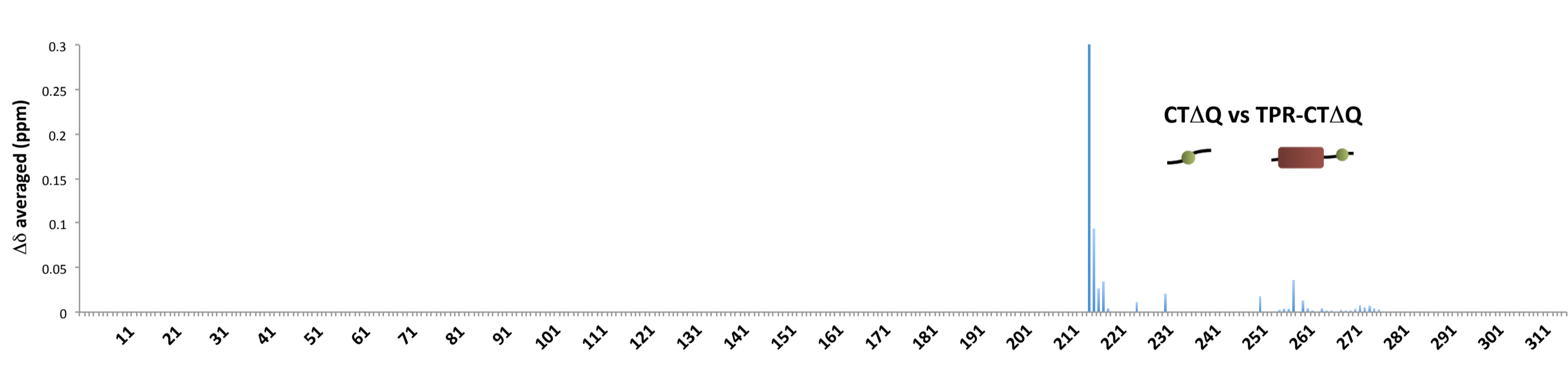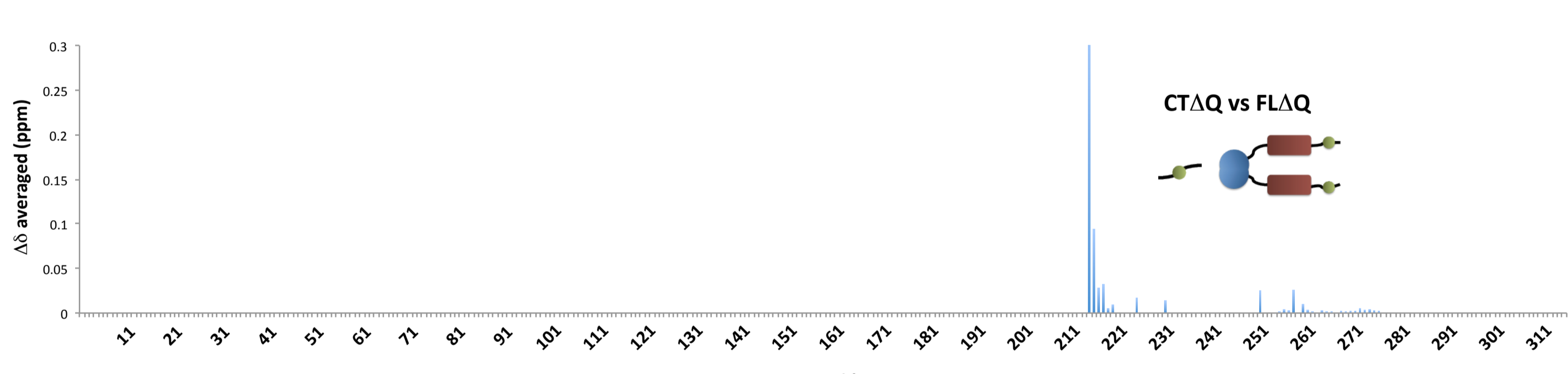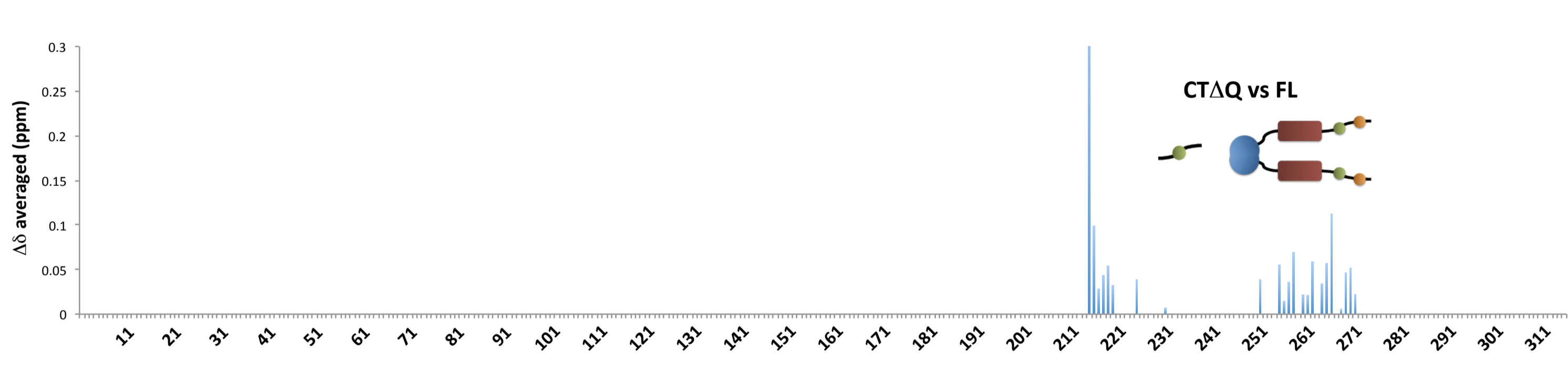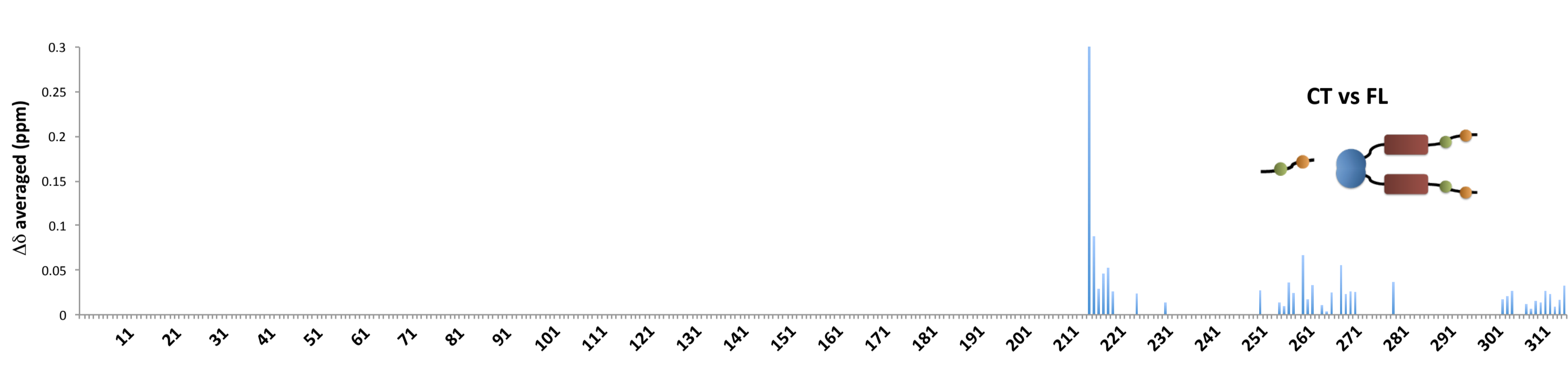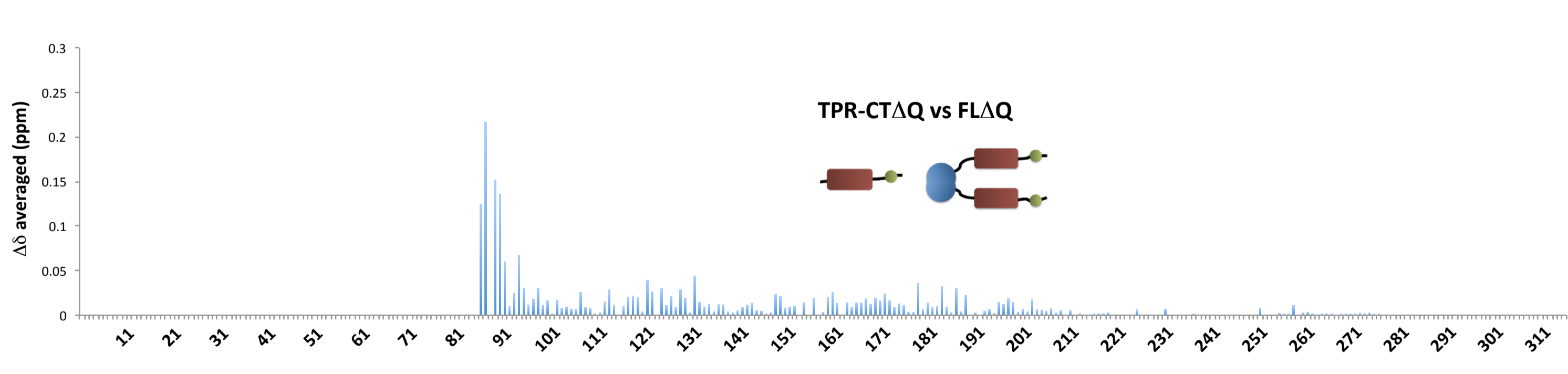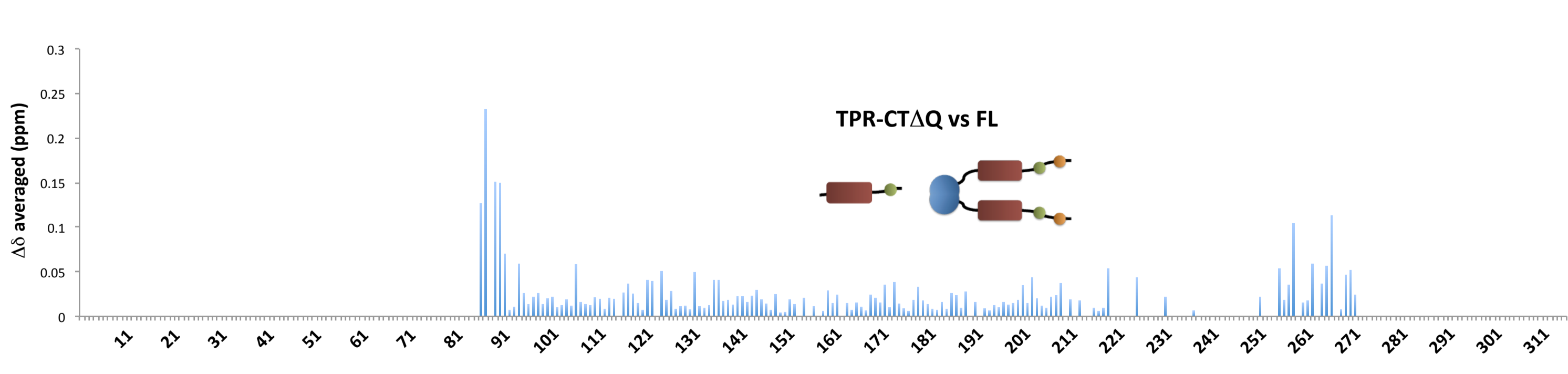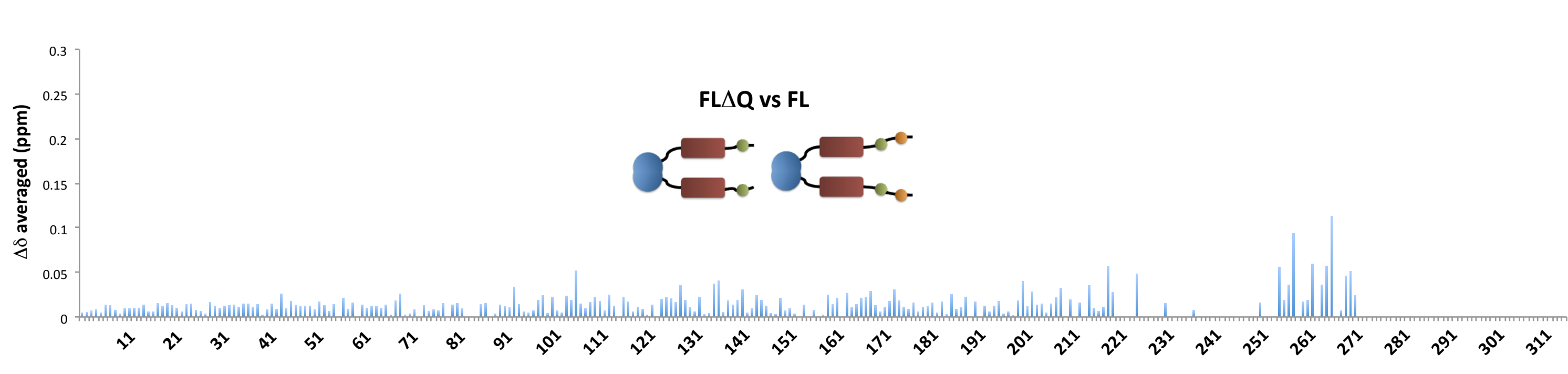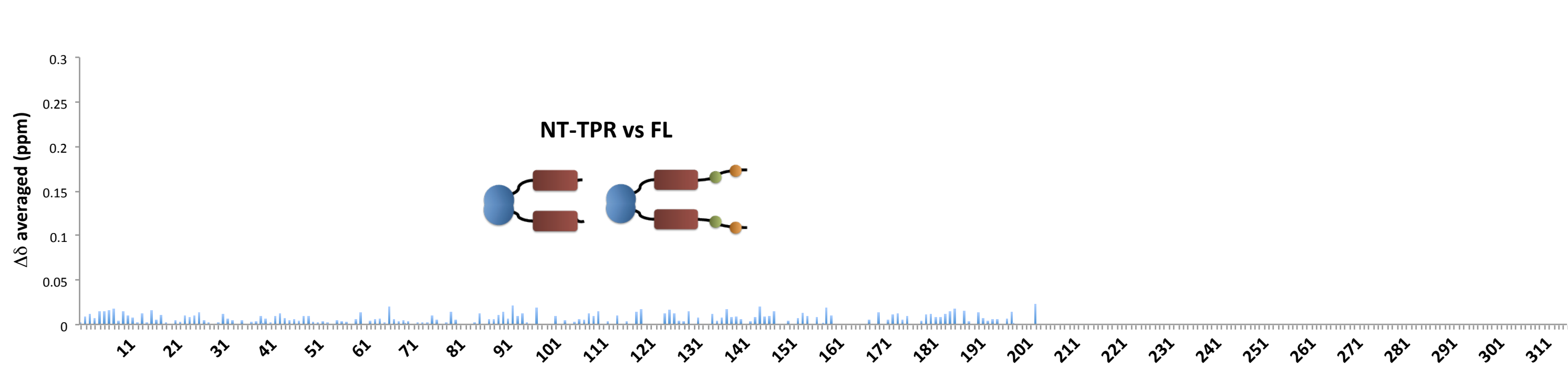

Supplement: Supplementary file 8 — Figure S8. Mass spectrometry analysis of SGTA CT construct, showing that the protein is not covalently modified. Expected molecular weight = 10,877 Da. (PDF 2643 kb) [file 12915_2018_542_MOESM8_ESM.pdf]

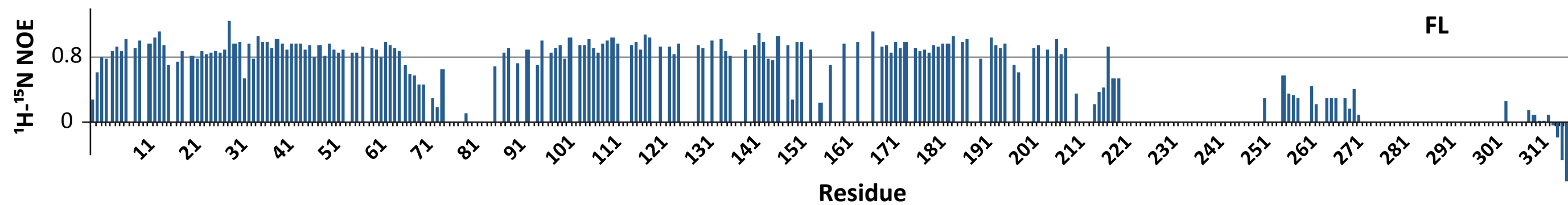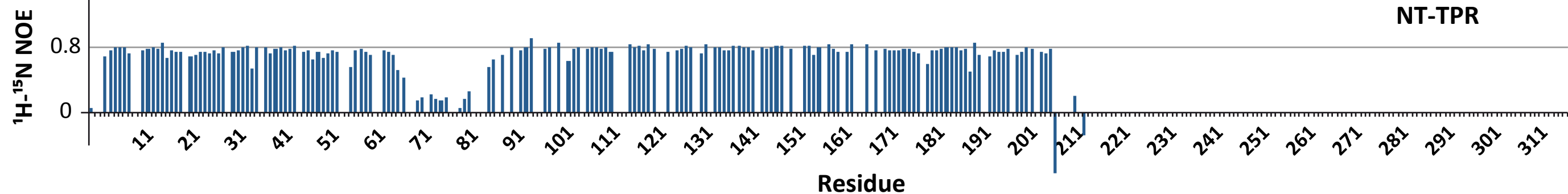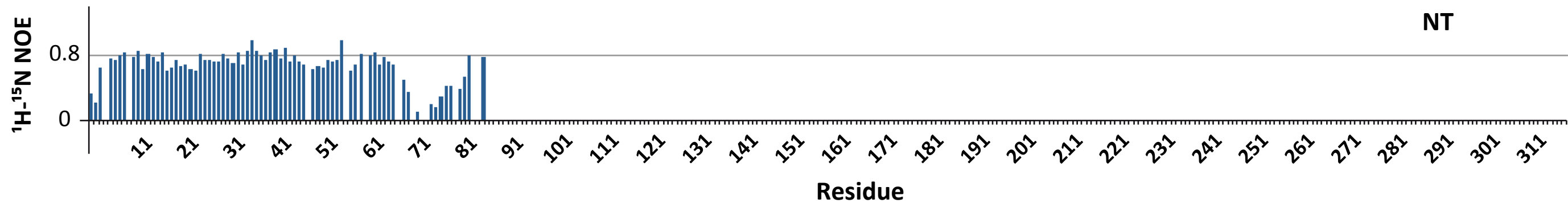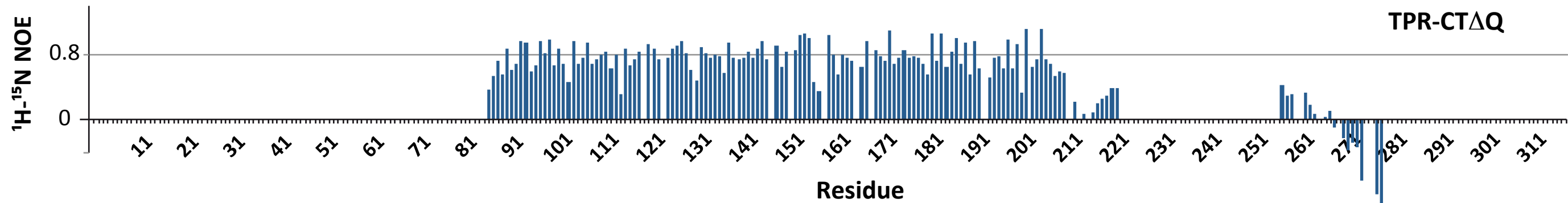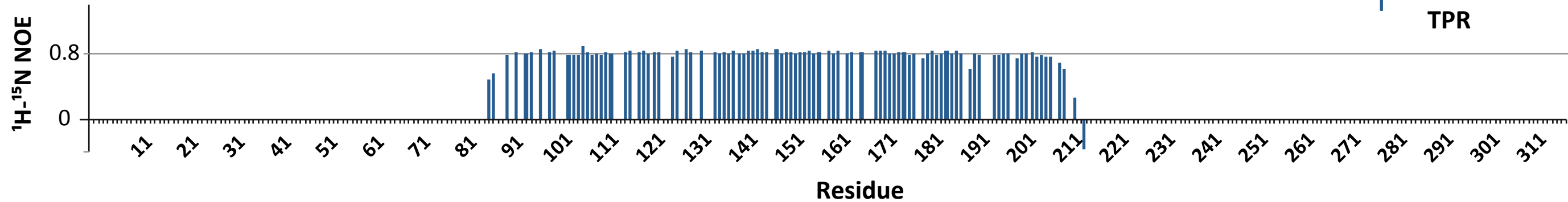

Supplement: Supplementary file 9 — Figure S9. Overlaid 1H-15N HSQC spectra of different SGTA constructs under the same conditions. (A) SGTA FL (black), NT (blue), TPR (red), and CT (green) proteins. (B) SGTA NT-TPR (black), NT (blue), and TPR (red) constructs. (C) SGTA TPR-CTΔQ (black), TPR (red), and CTΔQ (green) versions. (D) SGTA CT (black) and CTΔQ constructs (green). (PDF 271 kb) [file 12915_2018_542_MOESM9_ESM.pdf]

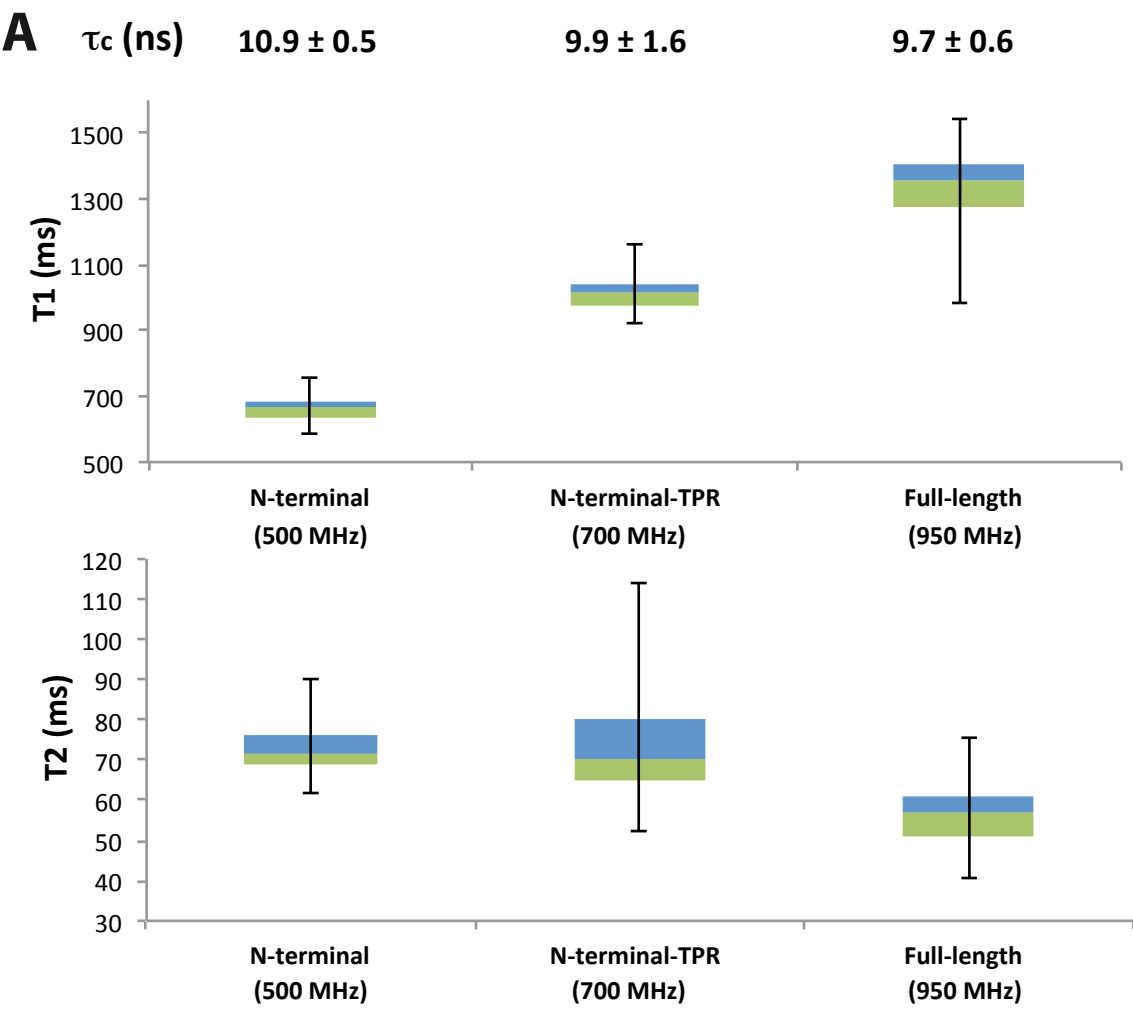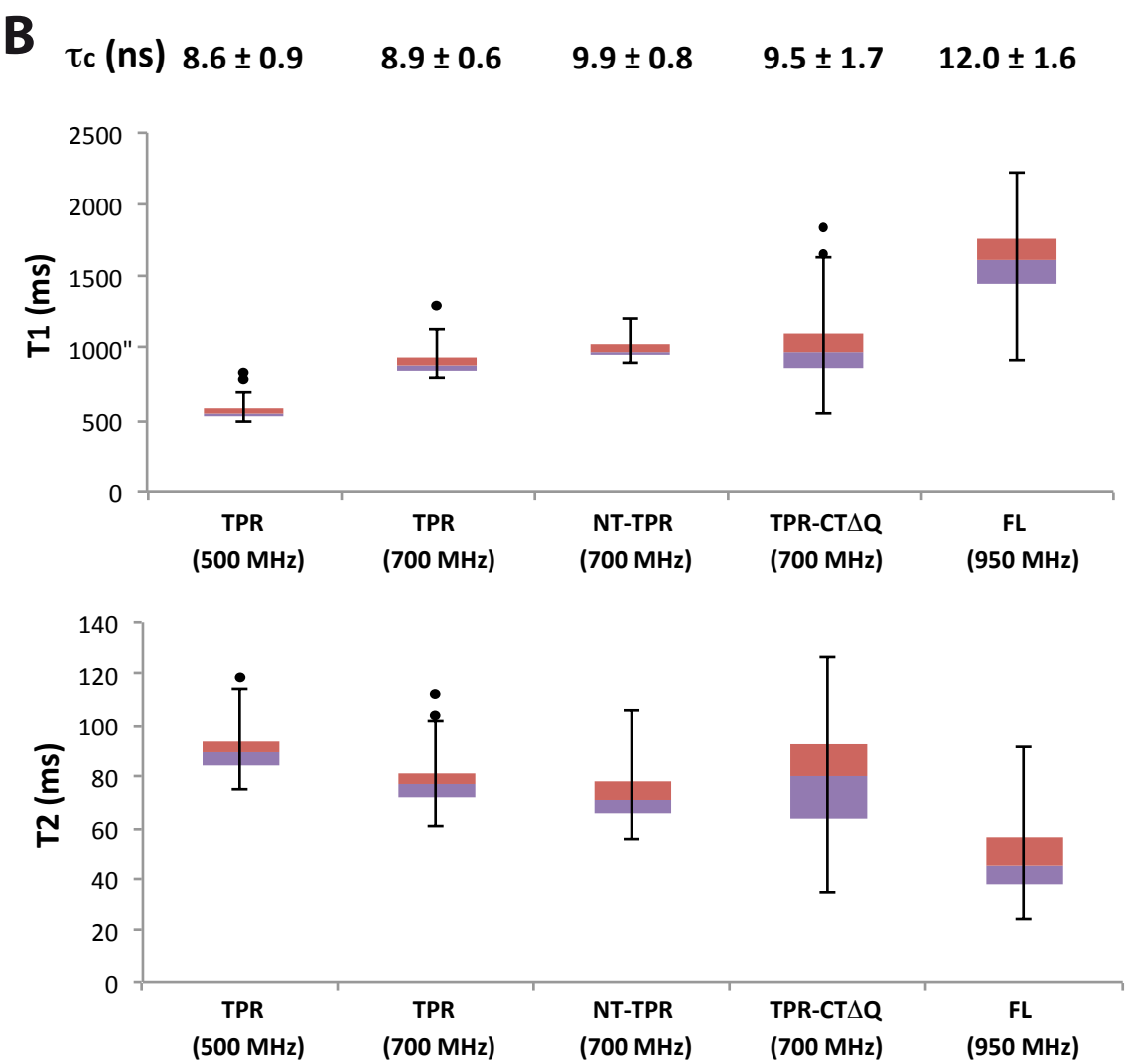

Supplement: Supplementary file 10 — Figure S10. 15N - {1H} heteronuclear NOE measurements of different SGTA constructs; in all cases, the domain boundaries and linker regions are similar. (PDF 142 kb) [file 12915_2018_542_MOESM10_ESM.pdf]

**A**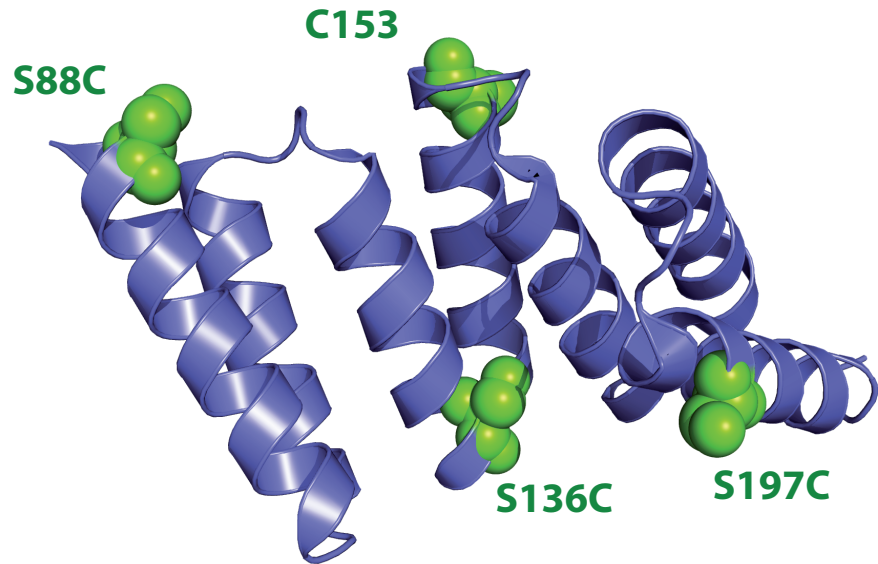**B**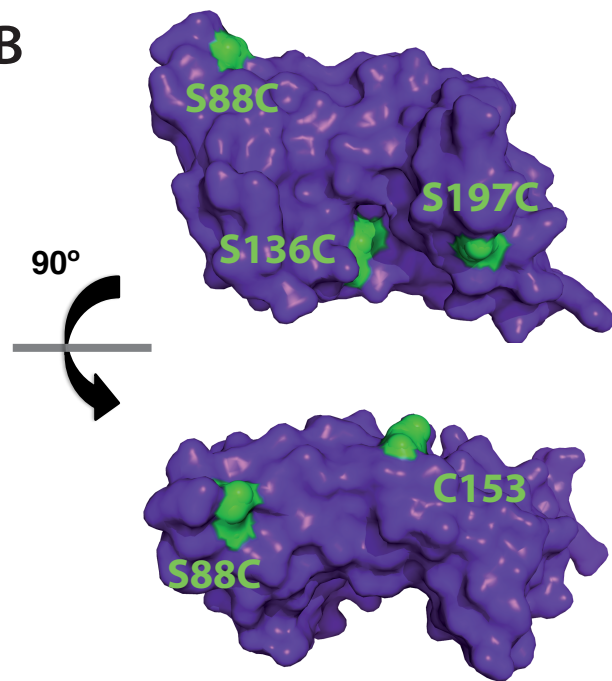

Supplement: Supplementary file 12 — Figure S11. 15N NMR relaxation analysis of N-terminal (A) and TPR (B) domains in different SGTA constructs. NT = residues 5–65: in construct NT, n = 57; in construct NT-TPR, n = 45; and in construct FL, n = 37. TPR = residues 87–205: in construct TPR, n = 97; in construct NT-TPR, n = 81; in construct TPR-CTΔQ, n = 92; and in construct FL n = 40. Boxplots show the T1 and T2 values obtained for residues of each domain presenting the median, the interquartile range (colored boxes), the maximum and minimum values (segments with whiskers), and the outliers (dots); the correlation times (shown above) were calculated using the averaged value and the standard deviation as set in the “Methods” section. (PDF 4187 kb) [file 12915_2018_542_MOESM12_ESM.pdf]

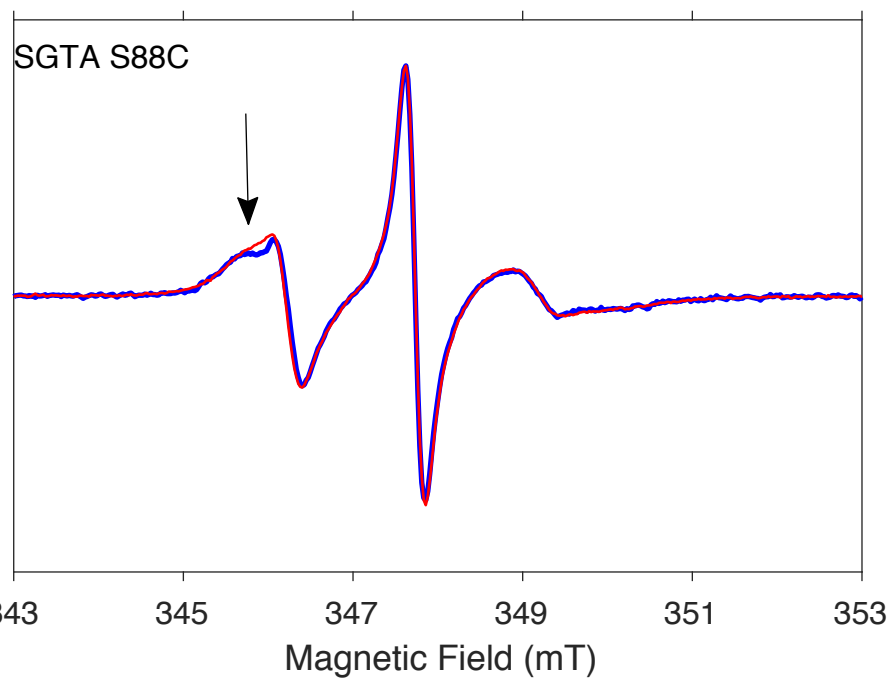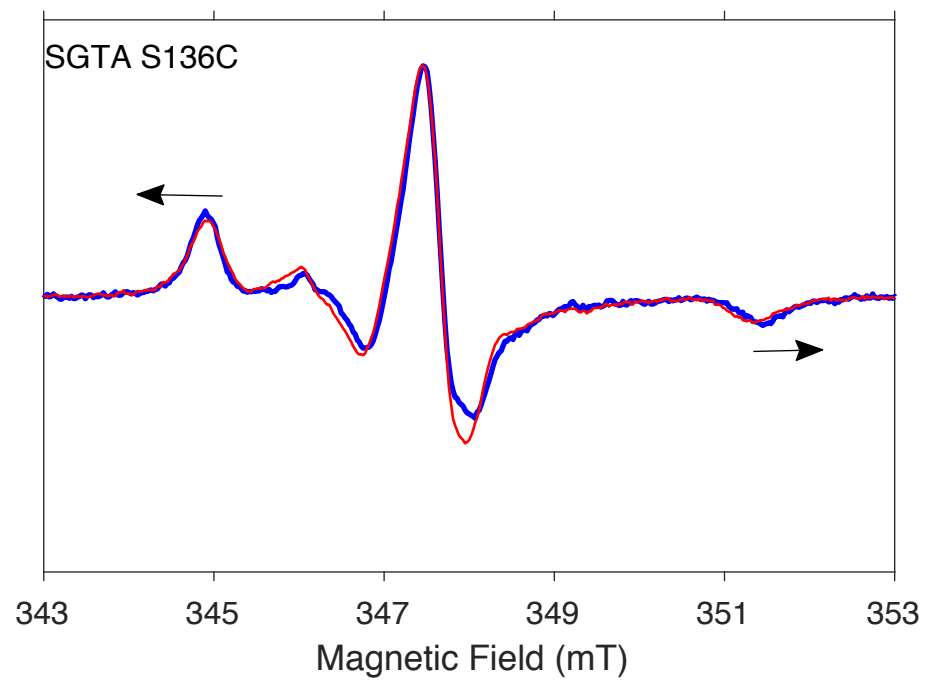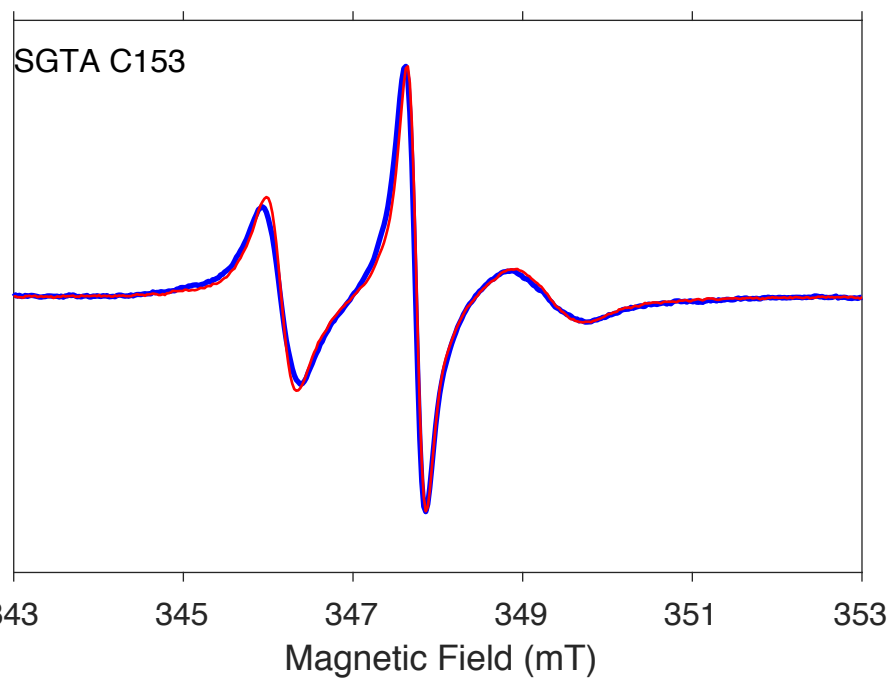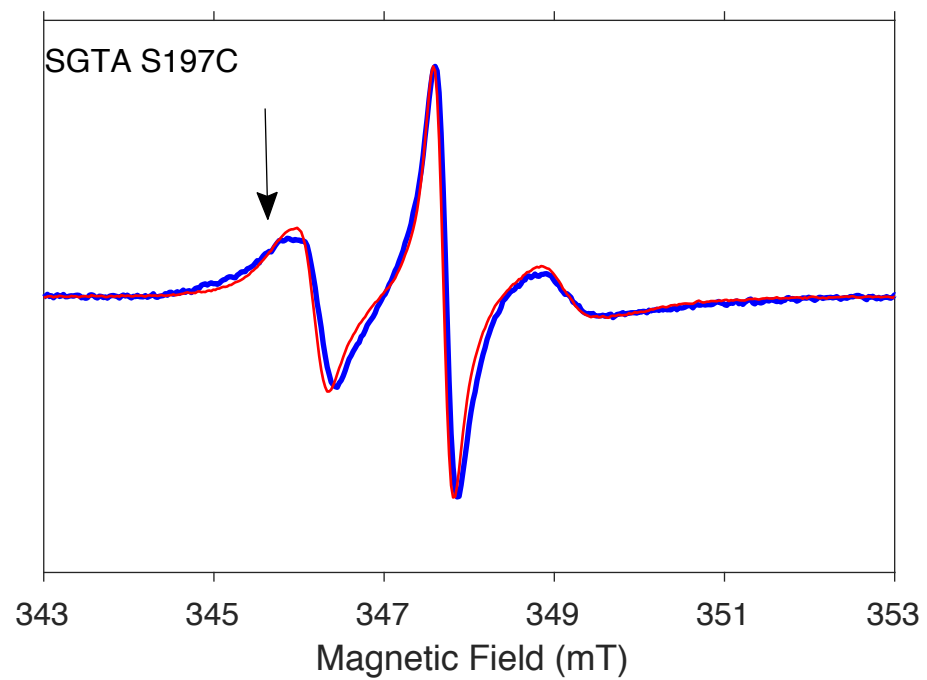

Supplement: Supplementary file 13 — Figure S12. Selected mutations in the TPR domain for MTSL labelling in the EPR experiments. (A) Cartoon representation of the TPR domain with the four positions depicted as green balls. (B) Surface representation of the TPR domain with the four residues colored in green. Notice that the S136C mutation is inside the TPR groove. (PDF 129 kb) [file 12915_2018_542_MOESM13_ESM.pdf]

i)

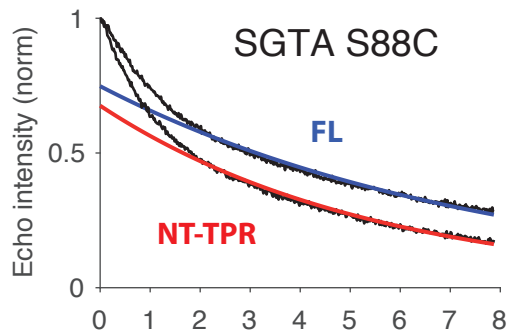

ii)

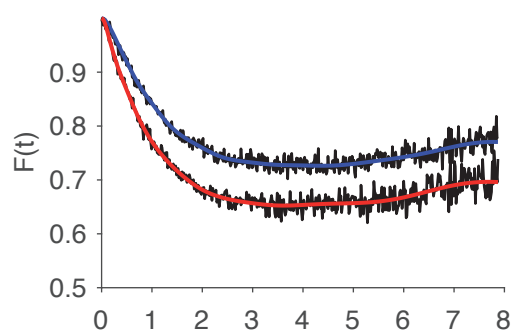

iii)

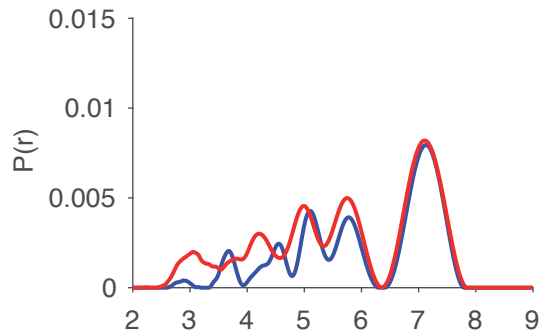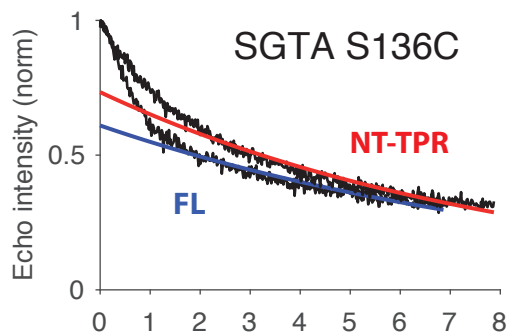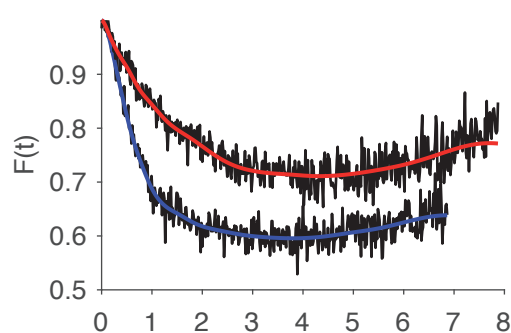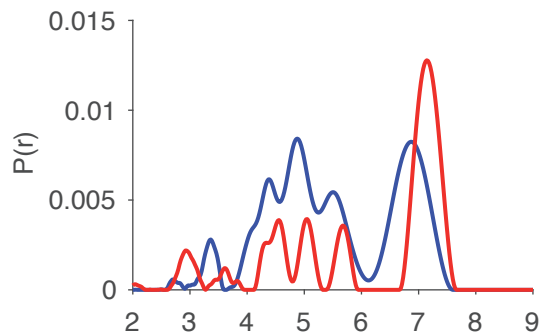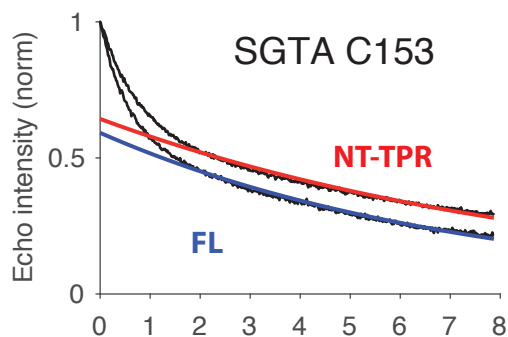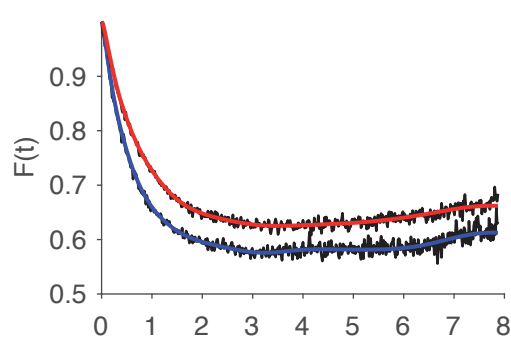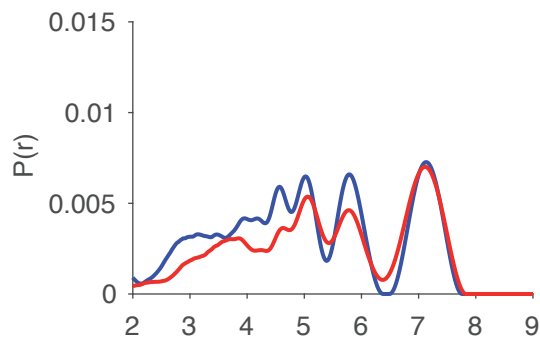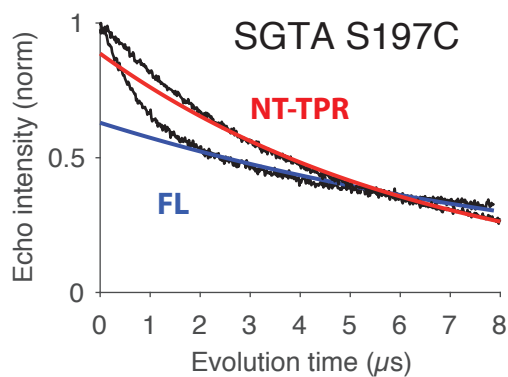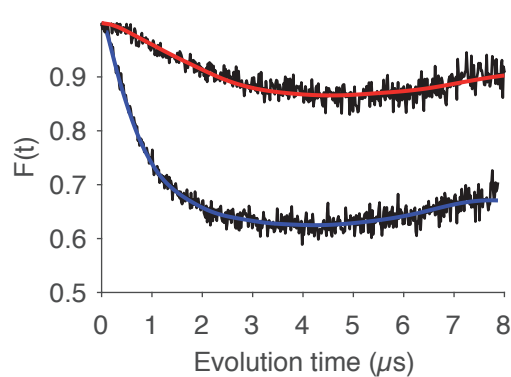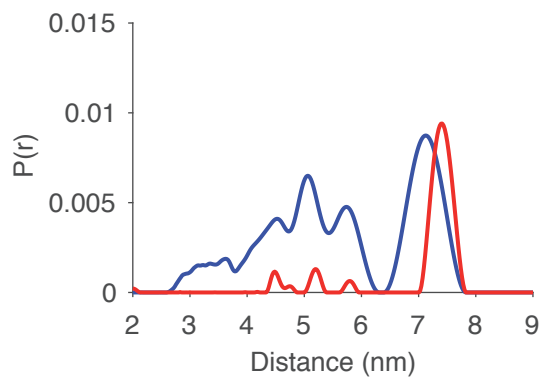

Supplement: Supplementary file 14 — Figure S13. CW-EPR spectra of the FL and C-terminal deleted (NT-TPR) SGTA proteins. Room Temperature CW-EPR spectra for SGTA FL mutants (blue lines) and corresponding NT-TPR constructs (red lines). CW-EPR spectra mainly provide information about the mobility of the spin labels and thus about the local environment of the labeled residues. Spectra of the FL protein are slightly broader than those corresponding to the C-terminal deletion (NT-TPR), as highlighted by arrows in the figure, suggesting that the absence C-terminal domain increase the overall mobility of the TPR domain. (PDF 559 kb) [file 12915_2018_542_MOESM14_ESM.pdf]

40°C  
35°C  
30°C  
25°C  
20°C  
15°C  
10°C  
5°C

$^{15}\text{N}$  (ppm)

105  
110  
115  
120  
125  
130

$^1\text{H}$  (ppm)

9

8

7

6

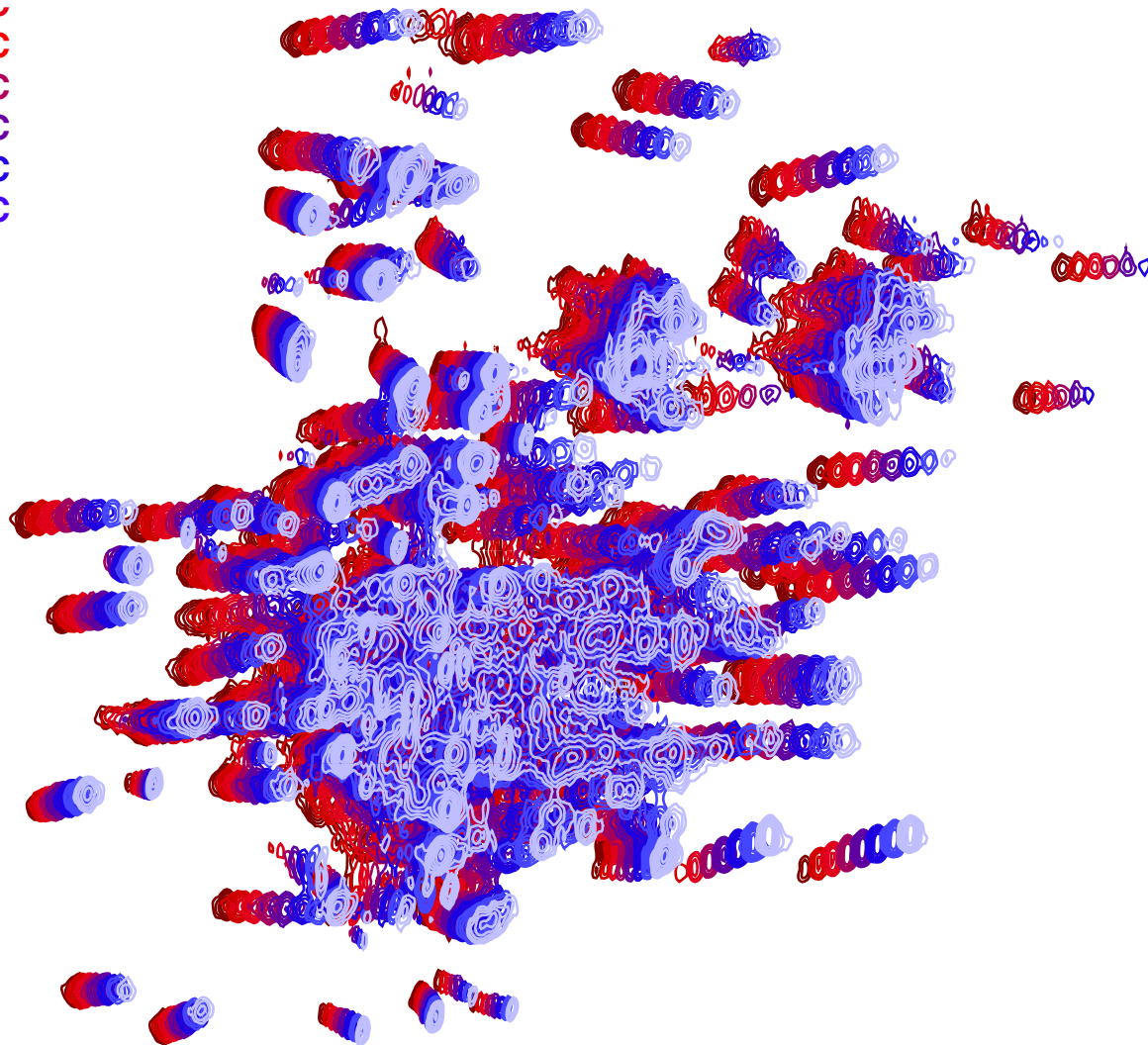

Supplement: Supplementary file 15 — Figure S14. DEER measurements and distances determined for FL and NT-TPR SGTA constructs spin-labeled in the four SGTA mutants. (i) Primary frequency-domain DEER data. (ii) Background-corrected dipolar evolution data (black lines) and corresponding fits obtained through DeerAnalysis2016 [49] by Tikhonov regularization. (iii) Distance distributions obtained by Tikhonov regularization. Blue fits: FL SGTA; red fits: NT-TPR construct. (PDF 3125 kb) [file 12915_2018_542_MOESM15_ESM.pdf]
