# Supplementary material for: Structural complexity of the co-chaperone SGTA: a conserved C-terminal region is implicated in dimerization and substrate quality control
Source: BMC Biol. 2018 Jul 11;16:76. doi: 10.1186/s12915-018-0542-3 (PMC6042327; doi:10.1186/s12915-018-0542-3)
Supplement: Supplementary file 4 — Figure S4. Overlaid 1H-15N HSQC spectra of TPR-CT∆Q SGTA at a range of temperatures from 5 °C (gray-blue) to 40 °C (maroon). (PDF 3684 kb) [file 12915_2018_542_MOESM4_ESM.pdf]

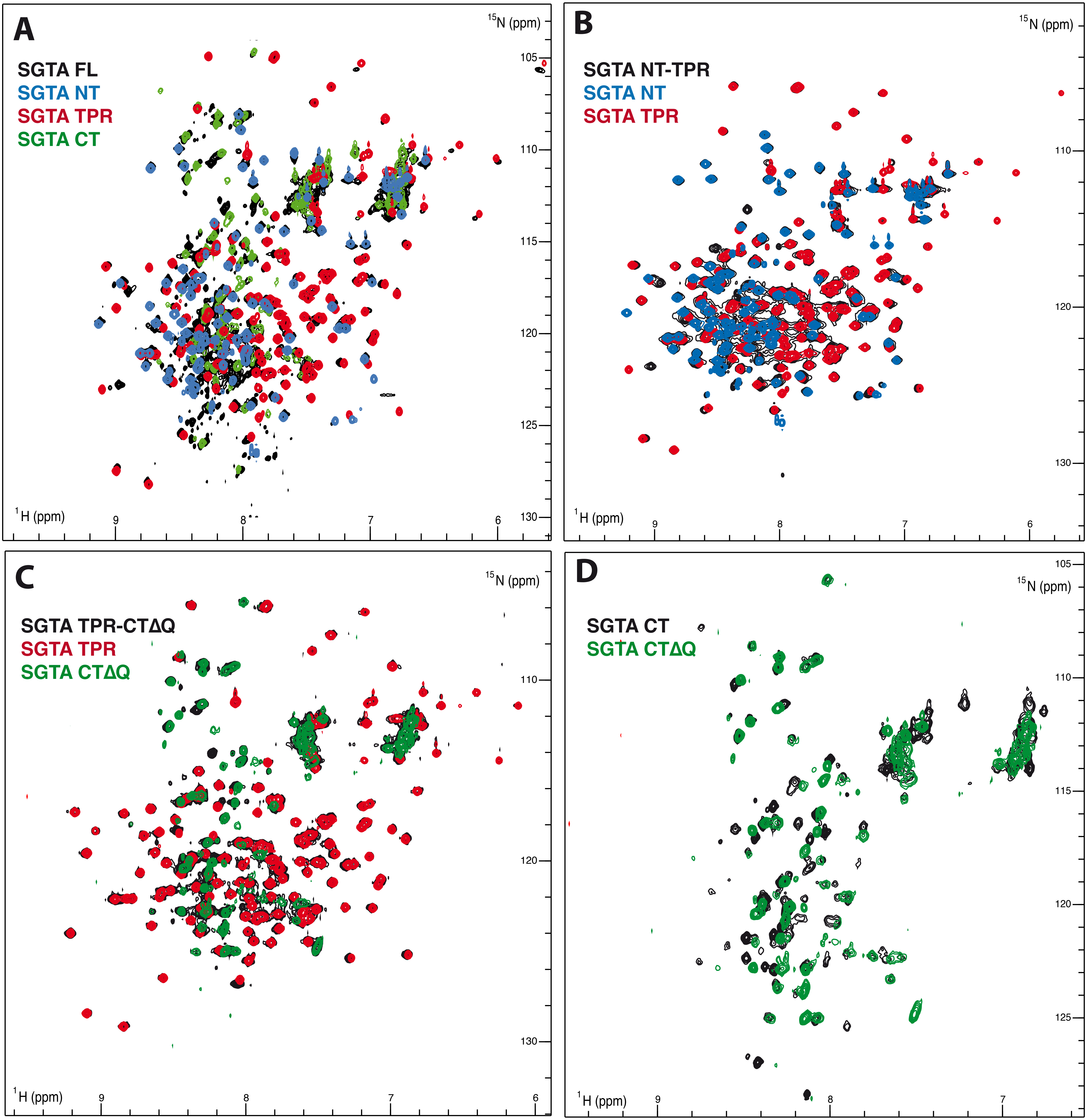
A
SGTA FL
SGTA NT
SGTA TPR
SGTA CT
B
SGTA NT-TPR
SGTA NT
SGTA TPR
C
SGTA TPR-CTΔQ
SGTA TPR
SGTA CTΔQ
D
SGTA CT
SGTA CTΔQ
15N (ppm)
1H (ppm)
