# Supplementary material for: Structural complexity of the co-chaperone SGTA: a conserved C-terminal region is implicated in dimerization and substrate quality control
Source: BMC Biol. 2018 Jul 11;16:76. doi: 10.1186/s12915-018-0542-3 (PMC6042327; doi:10.1186/s12915-018-0542-3)
Supplement: Supplementary file 11 — Table S1. Detailed SAXS data collection and derived parameters for FL and NT-TPR constructs of SGTA. (DOCX 46 kb) [file 12915_2018_542_MOESM11_ESM.docx]

**Table S1**

Detailed SAXS Data collection and derived parameters for FL and NT-TPR constructs of SGTA.

Abbreviations: *M_r_*: molecular mass; *R_g_*: radius of gyration; *D_max_*: maximal particle dimension; *V_p_*: Porod volume.

|  | ENTH |  | |  |  |
| --- | --- | --- | --- | --- | --- |
| **Data collection**  **parameters** |  | | |  |  |
| Instrument | EMBL P12 beam line (PETRA-III, DESY, Hamburg) | | |  |  |
| Beam geometry | 0.2 x 0.12 mm^2^ | | |  |  |
| Wavelength (Å) | 1.24 | | |  |  |
| *s* range (Å^-1^)^a^ | 0.01-0.46 | | |  |  |
| Exposure time (s) | 1 (20×0.05 s) | | |  |  |
| Concentration range (mg/mL) | 0.6-8.8 mg/ml |  |  |  |  |
| Temperature (K) | 298 |  |  |  |  |
| **Structural parameters^b^** | FL | NterTPR | |  |  |
| *I(0)* (cm^-1^) [from *p(r)*] | 17000 ± 60 | 10980 ± 70 | |  |  |
| *R_g_* (nm) [from *p(r)*] | 4.5 ± 0.3 | 3.8 ± 0.1 | |  |  |
| *I(0)* (cm^-1^) (from Guinier) | 16855 ± 62 | 10843 ± 16 | |  |  |
| *R_g_* (nm) (from Guinier) | 4.2 ± 0.2 | 3.6 ± 0.1 | |  |  |
| *D_max_* (nm) | 18 ± 1 | 14 ± 1 | |  |  |
| Porod volume estimate (Å^3^) | 153.0 ± 1.2 | 64.3 ± 0.3 | |  |  |
| Dry volume calculated from sequence (Å^3^)^c^ | 82.7 assuming dimer | | 57.1 assuming dimer | |  |
| **Molecular-mass determination** |  | | |  |  |
| *I(0)* (cm^-1^) BSA (66 kDa) | 19568 ± 25 | |  |  |  |
| Molecular mass *M*_r_ (kDa) [from *I(0)*] | 57 ± 2 | 37 ± 2 | |  |  |
| Molecular mass *M*_r_ (kDa) [from Porod volume (*V_p_/1.6)*] | 65 ± 5 | 41 ± 4 | |  |  |
| Calculated monomeric *M*_r_ from sequence (kDa) | 34.2 | | 23.6 | | |
| **Software employed** |  | | |  |  |
| Primary data reduction | RADAVER | | |  |  |
| Data processing | PRIMUS/Qt | | |  |  |
| Ab initio analysis | DAMMIF | | |  |  |
| Validation and averaging | DAMAVER | | |  |  |
| Ensemble Optimization | EOM 2.0 | | |  |  |
| Rigid-body modeling | SASREF | | |  |  |
| Computation of model intensities | CRYSOL | | |  |  |
| 3D graphics representations | PyMOL, UCSF Chimera | | |  |  |

^a^Momentum transfer *s* = 4πsin(θ)/λ. ^b^Values reported for 1.5 mg mL^-1^. Dry volume determined using the server: <http://www.basic.northwestern.edu/biotools/proteincalc.html>
